# 4E-BP1 differentially regulates translation of a subset of human mRNAs

**DOI:** 10.1016/j.jbc.2025.110976

**Published:** 2025-11-26

**Authors:** Mehreen Mahbub, Baishakhi Saha, Dixie J. Goss

**Affiliations:** 1 Department of Chemistry, Hunter College, City University of New York, New York, USA; 2 PhD. Program in Biochemistry, The Graduate Center of the City University of New York, New York, USA; 3 PhD. Program in Chemistry, The Graduate Center of the City University of New York, New York, USA

**Keywords:** cap-independent translation initiation, 5′ cap-independent translation enhancer (CITE), internal ribosome entry site (IRES), eIF4GI_557-1599_, eIF4E, 4E-BP1, eIF4A

## Abstract

Elevated levels of eukaryotic initiation factor 4E-binding protein 1 (4E-BP1) influence cap-independent translation by forming a complex with eukaryotic initiation factor 4E (eIF4E) (eIF4E•4E-BP1). Certain mRNAs—including those encoding hypoxia-inducible factor-1α (HIF-1α), fibroblast growth factor-9 (FGF-9), and two isoforms of tumor suppressors, p53 (p53_A_, and p53_B_)—contain structured 5′untranslated regions (UTRs) that enable translation either *via* cap-independent translation enhancer (CITE)-like or internal ribosomal entry site (IRES)-like mechanisms. However, how 4E-BP1 modulates these mechanisms remains unclear. Using fluorescence-based anisotropy assays, we showed that the eIF4E•4E-BP1 binds more tightly to the 5′ m^7^G-cap of these mRNAs than eIF4E alone. Luciferase reporter assays further demonstrated that 4E-BP1 inhibits translation of CITE-like (HIF-1α, and p53_A_) more effectively than IRES-like (FGF-9, and p53_B_) mRNAs. Although binding affinity of eIF4E•4E-BP1 to each of these mRNAs increased, only CITE-like mRNA translation was significantly inhibited. Importantly, eIF4GI_557-1599_ and its binding partner eIF4A, overcomes this inhibition selectively for CITE-like mRNAs, while eIF4E alone has only partial effects. In contrast, IRES-like mRNAs, despite binding to eIF4E•4E-BP1, exhibited only a modest translational repression and were minimally affected by eIF4GI_557-1599_•eIF4A or eIF4E. These findings reveal that 4E-BP1 selectively represses cap-independent translation in a transcript-specific manner. Moreover, fluorescence anisotropy revealed that 4E-BP1 enhances eIF4GI_557-1599_ recruitment to certain mRNAs, potentially facilitating 43S pre-initiation complex (43S PIC) assembly during stress. These results provide new mechanistic insights into selective translational control with implications for stress response and cancer progression *via* non-canonical translation.

Protein translation initiation is a complex and highly orchestrated process essential for maintaining cellular homeostasis and is a critical target in cancer development and tumor progression. This translation regulation involves the recruitment of translational machinery to mRNAs, through specific structural features, most notably the m^7^G-cap at the 5′ end of the mRNA and is vital to cellular physiology. Its disruption contributes to disease (1). Under basal cellular conditions, eukaryotic initiation factor 4E (eIF4E) availability governs cap-dependent translation initiation and serves as a rate-limiting step (2, 3). Its availability is tightly regulated by eukaryotic initiation factor 4E-binding protein 1 (4E-BP1), a stable (4, 5) downstream effector of mechanistic target of rapamycin (mTOR) activity (6, 7, 8, 9) that binds eIF4E to form a inhibitory complex (hereafter referred to as eIF4E•4E-BP1), blocking translation initiation until 4E-BP1 is phosphorylated. A fundamental distinction in translation mechanisms lies between cap-dependent and eIF4E-independent translation initiation, the latter allowing cells to bypass reliance on eIF4E when cap-dependent translation is impaired. eIF4E-independent translation is particularly important under stress conditions such as tumor hypoxia, viral infection, DNA damage, and nutrient deprivation (10, 11, 12, 13, 14). In these contexts, certain mRNAs, including those encoding stress response proteins, exploit these non-canonical mechanisms to recruit the ribosome independent of eIF4E recognition of the m^7^G-cap.

During cap-dependent translation initiation, 4E-BP1 undergoes hyperphosphorylation by the kinases of the mTOR pathway. Hyperphosphorylated 4E-BP1, with critical phosphorylation sites at Thr37/Thr46, Ser65, and Thr70 (4, 15), dissociates from eIF4E. This dissociation is often facilitated by polyubiquitination, which reduces the affinity of 4E-BP1 for eIF4E (4). The release of eIF4E from the eIF4E•4E-BP1 allows eIF4E to bind to the 5′ cap of mRNAs and recruit other eIFs to form the eukaryotic initiation factor 4F (eIF4F) complex near the 5′cap to initiate translation. The eIF4F complex, comprising of eIF4E (cap-binding subunit), eIF4A (mRNA helicase), and eIF4GI (scaffolding protein) (16), then facilitates the assembly of the 43S Pre-initiation complex (PIC) onto the 5′cap of mRNA and the machinery starts to move toward the start codon (AUG) to initiate translation (16, 17, 18). However, under unfavorable cellular conditions, mTOR inhibition leads to hypo-phosphorylation and subsequent activation of 4E-BP1 (7, 19, 20, 21). This process, likely mediated by protein phosphatase 2A (22), enables 4E-BP1 to bind to eIF4E, inhibiting translation of most mRNAs. However, certain mRNAs can bypass 4E-BP1 inhibition and utilize non-canonical translation, collectively termed as eIF4E-independent translation. While it is commonly assumed that 4E-BP1 sequesters eIF4E to inhibit this translation, we show that this is not the case, and the mechanism is more complex. This alternative mechanism of translation initiation relies on structured elements within the 5’ untranslated regions (5′UTRs), bypassing the requirement for the cap-binding protein, eIF4E (16, 23, 24).

During stress conditions, eIF4E-independent translation allows cells to reprogram their translation process to preserve cellular homeostasis by maintaining formation of key regulatory proteins. A key regulator of this process is 4E-BP1, which is overexpressed in ∼40% of cancers (25), including breast (26, 27, 28), gastrointestinal, esophageal, and lung adenocarcinoma cancers (29, 30, 31). Its overexpression is often associated with aggressive tumor behavior and poor patient prognosis (29, 31, 32). Elevated 4E-BP1 levels shift the translational landscape, selectively promoting the eIF4E-independent translation of mRNAs *via* highly stable structures within the 5′UTRs of specific mRNAs. Two such mechanisms are Internal Ribosome Entry Sites (IRES)-like and Cap-Independent Translation Enhancers (CITE)-like mechanisms, allowing translation to proceed independently of the 5′ cap (13, 33, 34, 35). IRES-like mechanisms require minimal initiation factors and translation is initiated through direct recruitment of the 43S PIC at or near the AUG (34, 36, 37), bypassing the need for the 5' cap (38). On the other hand, CITE-like mechanisms require a free 5′ end to recruit initiation factors and initiate translation *via* 43S PIC scanning (16, 34, 38). These structured elements within the 5′UTRs enable selective translation of specific mRNAs even under conditions where cap-dependent initiation is suppressed, a strategy also exploited by various plant mRNA viruses (39). While these mechanisms are functionally defined, the precise secondary structures or sequence motifs within these 5′ UTRs have not been well studied. Examples of mRNAs that utilize these mechanisms include fibroblast growth factor-9 (FGF-9), hypoxia-inducible factor-1α (HIF-1α), and two isoforms of p53 (p53_A_, and p53_B_) (34), all of which are translationally upregulated under hypoxic conditions, where cap-dependent translation is inhibited (34), and 4E-BP1 is overexpressed (25). These mRNAs were chosen because they represent functionally relevant transcripts that bypass cap-dependent translation inhibition under stress. FGF-9, and p53_B_ encoding mRNAs seem to use an IRES-like mechanism of translation initiation (38), whereas, HIF-1α, and p53_A_ encoding mRNAs utilize a CITE-like mechanism of translation.

Beyond its role in translation, ∼30% of 4E-BP1 is nuclear and sequesters eIF4E, limiting its cytoplasmic availability (40, 41). The remaining (∼70%) cytosolic 4E-BP1 is pivotal in facilitating a switch from cap-dependent to eIF4E-independent mRNA translation under hypoxia, promoting tumor angiogenesis and growth (25, 42). Despite its pivotal role in translation regulation, the specific mechanisms by which elevated 4E-BP1 levels influence IRES- and CITE-like translation initiation remain poorly understood. While previous studies have demonstrated that elevated 4E-BP1 enhanced the selective translation of IRES-containing mRNAs under hypoxia (43), its impact on CITE-like mRNAs in response to stress or altered eIF levels is unclear. One potential mechanism is highlighted in a study by Haizel S. *et al*. (34), which reported that a subset of 5′UTR-containing mRNAs, including FGF-9 (IRES-like) and HIF-1α (CITE-like), utilize the direct recruitment of eIF4GI_557-1599_ to their structured 5′UTRs to initiate translation. It is unclear whether eIF4E is sequestered by 4E-BP1 and is driven away from the translation initiation system, or whether, once sequestered, eIF4E in complex with 4E-BP1 (eIF4E•4E-BP1) remains bound to the 5′ cap of the mRNA, directing eIF4GI_557-1599_ to the 5′UTR to initiate translation. To address this knowledge gap, this study investigated how the mRNAs encoding HIF-1α, p53_A_, FGF-9, and p53_B_ bypass 4E-BP1-mediated translational repression and whether eIF4GI_557-1599_•eIF4A or eIF4E can restore translation in these contexts. This study aims to differentiate IRES-like from CITE-like mechanism of translation under conditions of high 4E-BP1 expression. To systematically investigate these mechanisms, well-characterized functional (m^7^G-) or non-functional (ApppG-) capped reporter mRNAs with structured 5′UTRs were used to perform *in-vitro* translation assays and fluorescence anisotropy binding studies. As control, a capped, unstructured β-actin-Luc mRNA was included, which relies strictly on cap-dependent translation, serving as a baseline for translational repression by 4E-BP1. By dissecting these mechanisms and providing quantitative binding data, these findings provide a foundation for targeting translation as a therapeutic strategy in cancer and other diseases characterized by dysregulated eIF4E-independent translation.

## Results

### 4E-BP1 enhances eIF4E binding to 5′UTRs of mRNAs

Previous research has suggested that 4E-BP1 sequesters eIF4E to prevent cap binding (44) while other researchers have suggested the eIF4E•4E-BP1 complex binds to the cap to block translation initiation (3, 45, 46, 47). To investigate these two possibilities, binding affinities of eIF4E, eIF4E•4E-BP1, and 4E-BP1 to m^7^G- or ApppG-capped 5′UTRs of HIF-1α, p53_A_, FGF-9, and p53_B_ encoding mRNAs were measured using a fluorescence-based anisotropy assay. Anisotropy values were normalized and plotted as a function of protein concentration.

**Figure 1:**
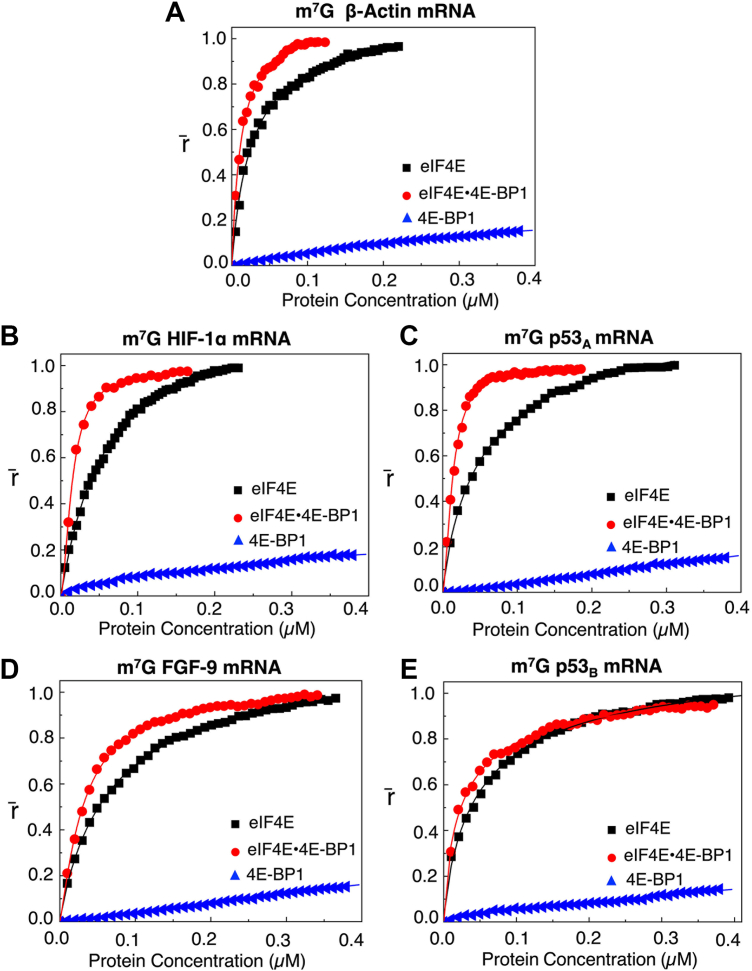
**Fluorescence anisotropy analyses of eIF4E and eIF4E•4E-BP1 binding to m^7^G-capped 5′UTRs of HIF-1α, p53_A_, FGF-9, and p53_B_ encoding mRNAs**. Normalized anisotropy changes (denoted as r̄) for the interaction of eIF4E (-▪-), eIF4E•4E-BP1 (), and 4E-BP1 () with 10 nM m^7^G-capped 5′UTR of (*A*) β-Actin (control), (*B*) HIF-1α, (*C*) p53_A_, (*D*) FGF-9 and (*E*) p53_B_ encoding mRNA transcripts fluorescein-labeled at 3′terminus, respectively. Data points corresponding to the average from three independent anisotropy measurements were normalized and plotted against protein concentration (μM). Curves represent Hill-equation fits, as described in materials and methods, used to calculate the corresponding K_D_ values.

These binding data demonstrated that eIF4E•4E-BP1 exhibits differential binding to these mRNAs (Table 1). The eIF4E•4E-BP1 bound m^7^G-capped mRNAs with significantly higher affinity than eIF4E alone. For the 5′UTR of HIF-1α mRNA, the equilibrium dissociation constant (K_D_) values decreased from (56 ± 1.5) nM with eIF4E alone to (15 ± 3.4) nM with the eIF4E•4E-BP1 (Table 1, Fig. 1*B*). Similarly, for the 5′UTR of p53_A_ mRNA, the K_D_ values decreased from (59 ± 7.5) nM to (13 ± 1.1) nM (Table 1, Fig. 1*C*). These results indicated that the eIF4E•4E-BP1 bound these mRNAs ∼4-5-fold more tightly than eIF4E alone, suggesting that 4E-BP1 enhanced eIF4Es mRNA-binding properties, potentially through structural (43) or allosteric regulation of eIF4E conformation (47). A more modest (∼2-fold) increase in affinity was observed for 5′UTRs of FGF-9, and p53_B_ mRNAs, with K_D_ values decreasing from (72 ± 1.6) nM to (32 ± 3.9) nM, and from (54 ± 2.3) nM to (28 ± 2.3) nM, respectively (Table 1, Fig. 1, *D* and *E*). Interestingly, 4E-BP1 alone did not bind any of these mRNAs, indicating that its mRNA association requires complex formation with eIF4E (Fig. 1, *B*–*E*). As a control, the 5′UTR of m^7^G-capped β-actin mRNA also showed increased binding affinity, with K_D_ values decreasing from (28 ± 1.8) nM to (12 ± 0.6) nM (Fig. 1*A*).

**Table 1:** Equilibrium dissociation constants K_D_ (nM) for protein-m^7^G-mRNA

Binding complex	m^7^G β-actin mRNA	m^7^G HIF-1α mRNA		m^7^G p^53_A_^ mRNA		m^7^G FGF-9 mRNA		m^7^Gp^53_B_^ mRNA	
	K_D_ (nM)	K_D_ (nM)	*χ*^2^	K_D_ (nM)	*χ*^2^	K_D_ (nM)	*χ*^2^	K_D_ (nM)	*χ*^2^
eIF4E	28 ± 1.8	56 ± 1.5	0.945	59 ± 7.5	0.957	72 ± 1.6	0.949	54 ± 2.3	0.996
eIF4E•4E-BP1	12 ± 0.6	15 ± 3.4	0.980	13 ± 1.1	0.979	32 ± 3.9	0.990	28 ± 2.3	0.963
4E-BP1	NSB	NSB		NSB		NSB		NSB	
eIF4GI_557-1599_ binding to mRNAs	NSB	22 ± 0.5	0.985	44 ± 1.0	0.987	20 ± 2	0.996	18 ± 3.1	0.984
eIF4GI_557-1599_ binding to mRNA• eIF4E•4E-BP1	NSB	15 ± 3.4	0.990	31 ± 5.9	0.960	24 ± 0.9	0.993	18 ± 0.3	0.995
eIF4GI_557-1599_• eIF4A binding to mRNA• eIF4E•4E-BP1	NSB	20 ± 1.3	0.995	33 ± 0.7	0.998	23 ± 1.3	0.991	21 ± 2.2	0.993
eIF4GI_557-1599_• eIF4A•ATP binding to mRNA•eIF4E•4E-BP1	NSB	21 ± 0.5	0.996	35 ± 1.3	0.995	24 ± 1.5	0.996	22 ± 1.1	0.998
eIF4GI_557-1599_ binding to mRNA bound eIF4E•4E-BP1-FITC-Peptide	NSB	21 ± 0.7	0.992	29 ± 1.3	0.985	32 ± 0.3	0.990	47 ± 0.7	0.993

K_D_ is the dissociation constant.

*χ*^2^ represents the goodness of fit. NSB, no significant binding.

**Figure 2:**
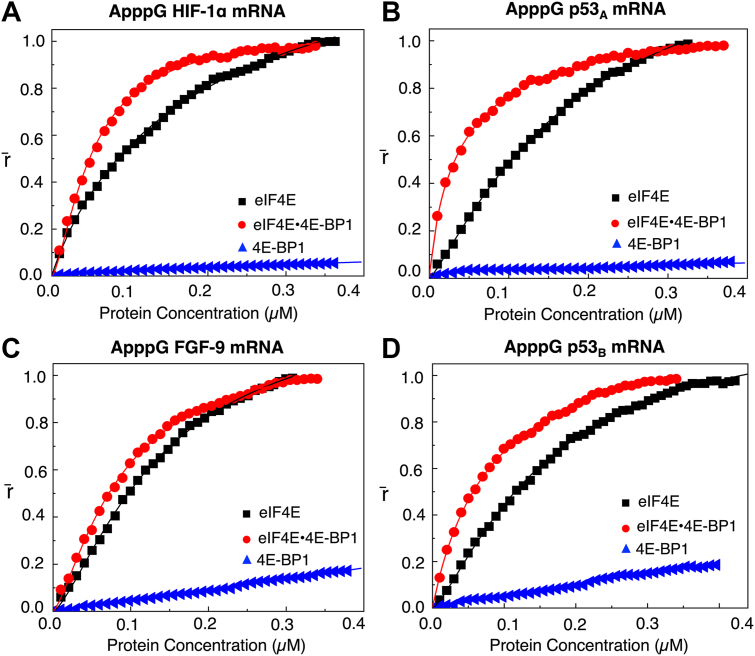
**Fluorescence anisotropy analyses of eIF4E and eIF4E•4E-BP1 binding to ApppG-capped 5′UTRs of HIF-1α, p53_A_, FGF-9, and p53_B_ encoding mRNAs**. Normalized anisotropy changes (denoted as r̄) for the interaction of eIF4E (-▪-), eIF4E•4E-BP1 (), and 4E-BP1 () with 10 nM ApppG-capped 5′UTR of (*A*) HIF-1α, (*B*) p53_A_, (*C*) FGF-9, and (*D*) p53_B_ encoding mRNA transcripts fluorescein-labeled at 3′terminus, respectively. Data points corresponding to the average from three independent anisotropy measurements were normalized and plotted against protein concentration (μM). Curves represent Hill-equation fits, as described in materials and methods, used to calculate the corresponding K_D_ values.

To further elucidate the mechanism of 4E-BP1-mediated translation regulation, protein interactions with non-functional ApppG-capped mRNAs were also examined. Like m^7^G-capped mRNA constructs, the eIF4E•4E-BP1 bound more tightly to ApppG-capped mRNAs than eIF4E alone. For example, binding affinities to the 5′UTRs of HIF-1α, and p53_A_ encoding mRNAs increased ∼4-fold, with K_D_ values decreasing from (199 ± 1.5) nM to (52 ± 0.5) nM, and from (186 ± 0.8) nM to (45 ± 1.6) nM, respectively (Table 2, Fig. 2, *A* and *B*). This enhanced binding affinity suggested that the eIF4E•4E-BP1 recognizes additional mRNA features beyond the m^7^G-cap. Although eIF4E is primarily known for its specificity to the m^7^G-cap (48, 49, 50, 51, 52), prior studies have shown it can also interact with other mRNA elements (48).

**Table 2:** Equilibrium dissociation constants K_D_ (nM) for protein-ApppG-mRNA

Binding complex	ApppG HIF-1α mRNA		ApppG p_53A_ mRNA		ApppG FGF-9 mRNA		ApppG p_53B_ mRNA	
	K_D_ (nM)	*χ*^2^	K_D_ (nM)	*χ*^2^	K_D_ (nM)	*χ*^2^	K_D_ (nM)	*χ*^2^
eIF4E	199 ± 1.5	0.999	186 ± 0.8	0.999	138 ± 4.6	0.991	159 ± 4.2	0.998
eIF4E•4E-BP1	52 ± 0.5	0.997	45 ± 1.6	0.998	87 ± 2.0	0.998	89 ± 3.5	0.990
4E-BP1	NSB		NSB		NSB		NSB	
eIF4GI_557-1599_ binding to mRNAs	67 ± 1.8	0.975	81 ± 1.3	0.977	28 ± 0.7	0.995	66 ± 1.7	0.990
eIF4GI_557-1599_ binding to mRNA• eIF4E•4E-BP1	25 ± 1.8	0.997	25 ± 1.1	0.994	13 ± 0.6	0.993	22 ± 0.8	0.995
eIF4GI_557-1599_• eIF4A binding to mRNA• eIF4E•4E-BP1	19 ± 0.6	0.994	20 ± 2.0	0.991	15 ± 0.3	0.994	28 ± 1.8	0.984
eIF4GI_557-1599_• eIF4A•ATP binding to mRNA• eIF4E•4E-BP1	24 ± 4.3	0.987	16 ± 0.8	0.992	18 ± 1.0	0.994	21 ± 1.1	0.990
eIF4GI_557-1599_ binding to mRNA bound eIF4E•4E-BP1-FITC-Peptide	30 ± 3.1	0.978	35 ± 0.5	0.994	25 ± 1.0	0.992	42 ± 2.0	0.996

K_D_ is the dissociation constant.

*χ*^2^ represents the goodness of fit. NSB, no significant binding.

Similarly, the presence of 4E-BP1-enhanced eIF4E’s binding to ApppG-capped 5′UTRs of FGF-9, and p53_B_ encoding mRNAs, leading to a ∼2-fold increase in binding affinity. In this case, K_D_ values decreased from (138 ± 4.6) nM to (87 ± 2.0) nM for 5′UTR of FGF-9 mRNA, and from (159 ± 4.2) nM to (89 ± 3.5) nM for 5′UTR of p53_B_ mRNA (Table 2, Fig. 2, *C* and *D*). Notably, the eIF4E•4E-BP1 consistently bound more tightly to functional m^7^G-capped than to non-functional ApppG-capped mRNAs across all constructs (Tables 1 and 2). This effect was especially pronounced in CITE-like mRNAs.

### 4E-BP1 differentially affects the translation of CITE-like mRNAs and IRES-like mRNAs

**Figure 3:**
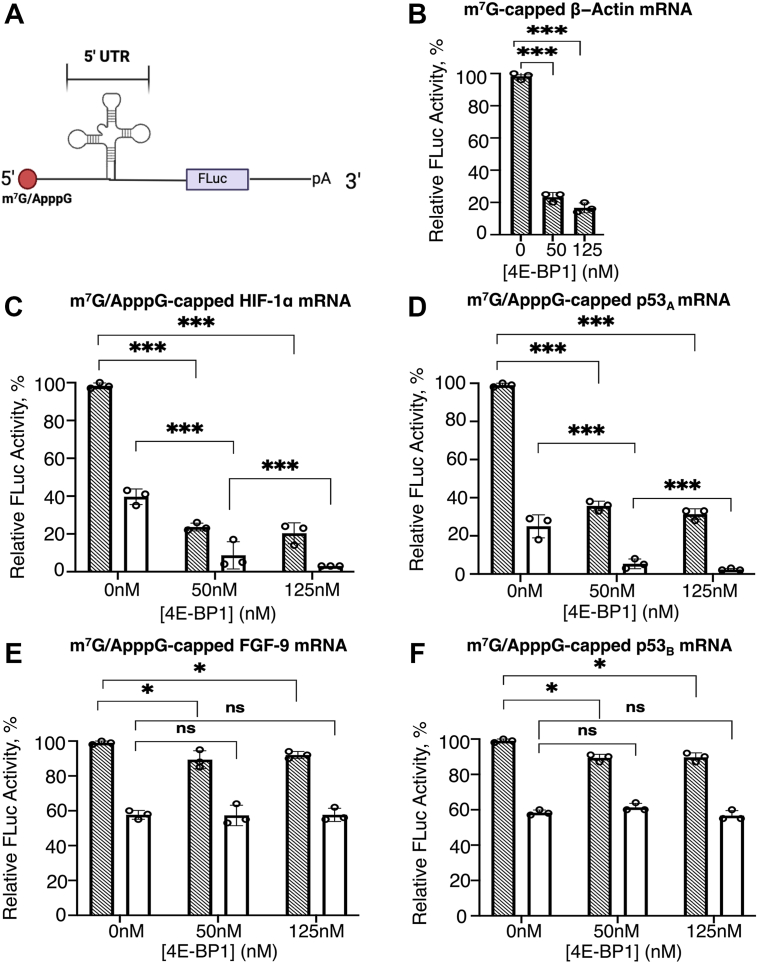
**4E-BP1 effects on the translation yields of m^7^G- and ApppG-capped-5′UTR-Luc mRNAs**. *A*, Cartoon representation of the mRNA reporter used for this study. Translation yields of (*B*) m^7^G-β-actin-UTR-Luc mRNA; Comparison of m^7^G- and ApppG-capped transcripts of (*C*) HIF-1α; (*D*) p53_A_, (*E*) FGF-9, and (*F*) p53_B_-UTR-Luc encoding mRNAs following addition of increasing concentrations of 4E-BP1. Relative luciferase activity was normalized to the respective controls (m^7^G-capped-UTR-Luc mRNA) for each reporter constructs with no 4E-BP1 added to non-depleted RRL. Diagonally *striped bar* (--), represents m^7^G-capped mRNAs; and *white bar* (--), represents ApppG-capped RNAs, respectively. Bar heights and error bars correspond to the average and standard deviations, respectively, of three independent luciferase activity measurements. Data were analyzed by two-tailed unpaired Student’s *t* test, where p represents the probability that differences occurred by chance: n.s, p = 0.12; ∗, p < 0.033; ∗∗, p = 0.002; ∗∗∗, p < 0.001.

In general, 4E-BP1 regulates cell proliferation and survival by modulating translation of specific mRNA subsets (53, 54). To investigate its role in eIF4E-independent translation of HIF-1α, p53_A_, FGF-9, and p53_B_-UTR-Luc mRNAs respectively, *in-vitro* translation assays were performed using nuclease-treated rabbit reticulocyte lysate (RRL) under cap-dependent translation conditions. β-actin-UTR-Luc mRNA, which is strictly cap-dependent under these conditions (34, 55), served as a control to validate the cap-dependency of our system. To corelate binding affinity of the eIF4E•4E-BP1 with its translational output, luciferase reporter mRNAs containing the respective 5′UTRs (UTR-Luc) were synthesized with either m^7^G- or ApppG-caps (Fig. 3*A*), the latter serving as a non-functional cap to protect the 5′ end from degradation. Although the eIF4E•4E-BP1 can associate with both cap structures, binding to m^7^G-capped mRNAs was significantly tighter (Tables 1 and 2, Fig. 1, *A*–*E*, and 2, *A*–*D*). For CITE-like mRNAs, the relative luciferase expression markedly decreased in response to increasing 4E-BP1 concentrations. As the concentration of 4E-BP1 increases, the relative expression level of m^7^G-capped HIF-1α-UTR-Luc mRNA declined to less than ∼20% of its maximum, while the ApppG-capped transcripts were reduced even further to ∼5%, relative to its m^7^G-capped analogs (Fig. 3*C*). Similarly, translation of m^7^G-capped p53_A_-UTR-Luc mRNA declined to less than ∼36%, while the ApppG-capped transcript was reduced even further to ∼3%, relative to its m^7^G-capped counterparts (Fig. 3*D*). Although ApppG-capped mRNAs followed a similar inhibition trend compared to m^7^G-capped analogs, their baseline expression was inherently lower than the m^7^G-capped analogs. These results aligned well with earlier reports indicating a direct correlation between binding affinity and translation efficiency (34, 56). Given that CITE-like mRNAs require a free 5′end (43) and likely 5′end scanning (33, 38, 57, 58), the tighter binding of the eIF4E•4E-BP1 than eIF4E alone near the 5′cap or critical regions of the mRNAs, may form a steric barrier that impedes 43S PIC recruitment and scanning (27), thereby inhibiting translation initiation and potentially preventing tumor formation (59).

Unlike CITE-like mRNAs, the translation efficiency of m^7^G-capped IRES-like mRNAs was only modestly affected by 4E-BP1. For m^7^G-capped FGF-9-UTR-Luc mRNA, the translation efficiency was slightly reduced to ∼85% in the presence of 4E-BP1 (Fig. 3*E*). Similarly, m^7^G-capped p53_B_-UTR-Luc mRNA showed a minor reduction to ∼87% (Fig. 3*F*). In contrast, the ApppG-capped versions of these mRNAs, while inherently less efficient than their m^7^G-capped counterparts, exhibited little to no further inhibition by 4E-BP1, even at 50 nM 4E-BP1 (Fig. 3, *E* and *F*). These findings suggest that ApppG-capped IRES-like mRNAs, despite lower basal level of translation, are largely resistant to 4E-BP1-mediated translation inhibition. This supports the notion that eIF4E sequestration by 4E-BP1 doesn’t block cap-independent translation driven by the 5′UTRs of FGF-9, and p53_B_ encoding mRNAs. This is consistent with prior research in HEK293 T cells under stress, where activation of endogenous 4E-BP1 didn’t significantly impair translation of IRES-containing viral mRNAs (38). For the control, m^7^G-capped-β-actin-UTR-Luc mRNA, translation efficiency was significantly reduced to ∼25% by 4E-BP1 (Fig. 3*B*), in line with prior findings showing a ∼2.5-fold reduction of cap-dependent translation upon 4E-BP1 overexpression (55, 60). Conversely, the ApppG-capped-β-actin mRNA transcript showed <2% activity compared to its m^7^G-capped counterpart (Fig. S1), confirming the inefficiency of cap-independent translation for strictly cap-dependent mRNAs (38, 55). Together, these data demonstrated that 4E-BP1 differentially regulated cap-independent translation, strongly inhibiting translation of CITE-like mRNAs that require a 5′end access, while exerting minimal effects on IRES-like mRNAs that initiate translation in a 5′end independent manner.

### 4E-BP1-4Ala and wild-type 4E-BP1 similarly inhibit cap-dependent mRNA translation under hypo-phosphorylated conditions

To address the possibility that the observed effect of 4E-BP1 on each subset of mRNAs was due to its hypo-phosphorylation during purification or the assay, the effects of wild-type 4E-BP1 and a 4E-BP1 mutant with Ala substitution at phosphorylation sites were compared. A dominant negative control, a 4E-BP1 mutant that is constitutively hypo-phosphorylated with four negative regulatory phosphorylation sites on 4E-BP1 mutated to Ala (T37 A, T46 A, S65 A, T70 A), referred to as 4E-BP1-4Ala (an exogenous non-phosphorylated form of 4E-BP1) (61) was used. This mutant is basically immune to being shut off by mTOR-mediated phosphorylation and therefore is very active at binding eIF4E regardless of cellular stress conditions. To assess its functional impact, the effects of increasing concentrations of 4E-BP1-4Ala and wild-type 4E-BP1 on the relative expression level of m^7^G-capped HIF-1α, p53_A_, FGF-9, and p53_B_-UTR-Luc mRNAs were compared. There were no significant changes in the expression levels of these mRNAs across comparable concentrations of the two proteins (4E-BP1-4Ala, and wild-type 4E-BP1) (Fig. S2, *A*–*D*). A similar trend was observed for ApppG-capped UTR-Luc mRNAs (Fig. S3, *A*–*D*). These findings indicated that wild-type 4E-BP1 remained hypo-phosphorylated under these experimental conditions, effectively mimicking the natural stress conditions necessary for eIF4E-independent translation initiation.

### eIF4E partially restored 4E-BP1-mediated inhibition of CITE-like mRNA translation but has minimal effect on IRES-like mRNAs

**Figure 4:**
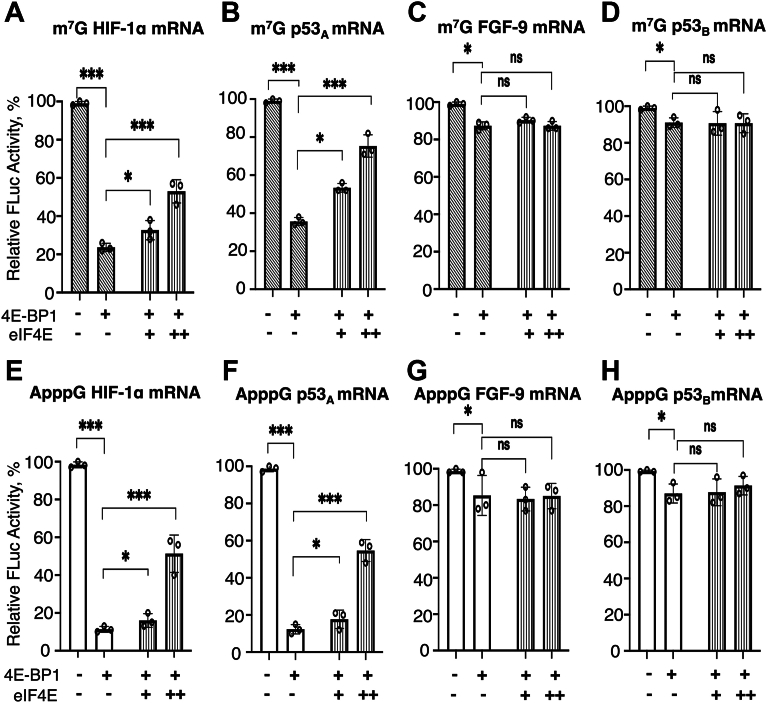
**Effect of eIF4E on the translation yields of m^7^G- and ApppG-capped HIF-1α, p53_A_, FGF-9, and p53_B_-5′UTR-Luc mRNA transcripts**. Translation yields of m^7^G-capped (*A*–*D*) and ApppG-capped (*E*–*H*) mRNA transcripts of HIF-1α-, p53_A_-, FGF-9-, and p53_B_-UTR-Luc mRNAs, respectively, following treatment of the RRL with 50 nM of 4E-BP1 and increasing concentrations of eIF4E. Conditions included 50 nM (denoted as +), and 150 nM (denoted as ++) of eIF4E. For m^7^G-capped mRNAs, 4E-BP1 alone (*diagonally striped bar*, --); increasing eIF4E concentration (*vertically striped bar*, --). For ApppG-capped mRNAs, 4E-BP1 alone (*white bar*, --); increasing eIF4E concentration (*vertically striped bar*, --).4E-BP1 was held constant at 50 nM in all conditions. Relative luciferase activity was normalized to the respective controls (ApppG-capped-UTR-Luc mRNA) for each reporter constructs with no 4E-BP1 added to RRL. Bar heights and error bars correspond to the average and standard deviations, respectively, of three independent luciferase activity measurements. Data were analyzed by two-tailed unpaired Student’s t test, where p represents the probability that differences occurred by chance: n.s, p = 0.12; ∗, p < 0.033; ∗∗, p = 0.002; ∗∗∗, p < 0.001.

Since 4E-BP1 sequesters eIF4E and limits its availability (43, 62), and eIF4E is crucial for cap-independent translation initiation (24), the effect of exogenously added eIF4E was tested in non-eIF4E-depleted cells while maintaining a constant 50 nM 4E-BP1 concentration. For m^7^G-capped CITE-like HIF-1α-UTR-Luc, and p53_A_-UTR-Luc mRNAs, increasing eIF4E concentrations (up to 150 nM), partially restored translation under constant 50 nM 4E-BP1 conditions. Translation of HIF-1α-UTR-Luc, and p53_A_-UTR-Luc mRNAs increased from <20% and <36% at 0 nM added eIF4E in the presence of 50 nM 4E-BP1 to ∼53% and ∼75% at 150 nM additional eIF4E, respectively (Fig. 4, *A* and *B*). Notably, eIF4E could not fully restore translation to baseline levels, likely because its binding affinity for these mRNAs is ∼4-fold lower compared to the eIF4E•4E-BP1 (Fig. 1, *B*–*C*, and 2, *A*–*B*). Similar results were observed with ApppG-capped constructs, where eIF4E partially restored the translation of CITE-like HIF-1α-UTR-Luc, and p53_A_-UTR-Luc mRNAs increasing expression from ∼15% and ∼17% (at 0 nM added eIF4E) to ∼54% and ∼56% (at 150 nM added eIF4E), respectively (Fig. 4, *E* and *F*). In contrast, for m^7^G-capped IRES-like FGF-9-UTR-Luc, and p53_B_-UTR-Luc mRNAs, the mild inhibition observed with 50 nM 4E-BP1 wasn’t improved by eIF4E addition, whether the transcripts were m^7^G- or ApppG-capped (Figure 4, *C*–*D* and *G*–*H*). These differences are likely due to unique 5′UTR features that influence eIF recruitment (63). For the control, m^7^G-capped-β-actin-UTR-Luc mRNA, addition of equimolar amount of 4E-BP1 and purified eIF4E significantly restored translation from ∼25% to ∼80% in the presence of 50 nM 4E-BP1 (Fig. S4*A*), consistent with prior reports (64, 65, 66). Overall, these results support a model in which eIF4E availability selectively modulates the translation of CITE-like mRNAs, while IRES-like mRNAs remain largely insensitive to eIF4E levels, likely due to their cap-independent mechanisms of 43S PIC recruitment.

### eIF4GI_557-1599_•eIF4A efficiently restored 4E-BP1-mediated inhibition of CITE-like mRNA translation but has minimal effect on IRES-like mRNAs

**Figure 5:**
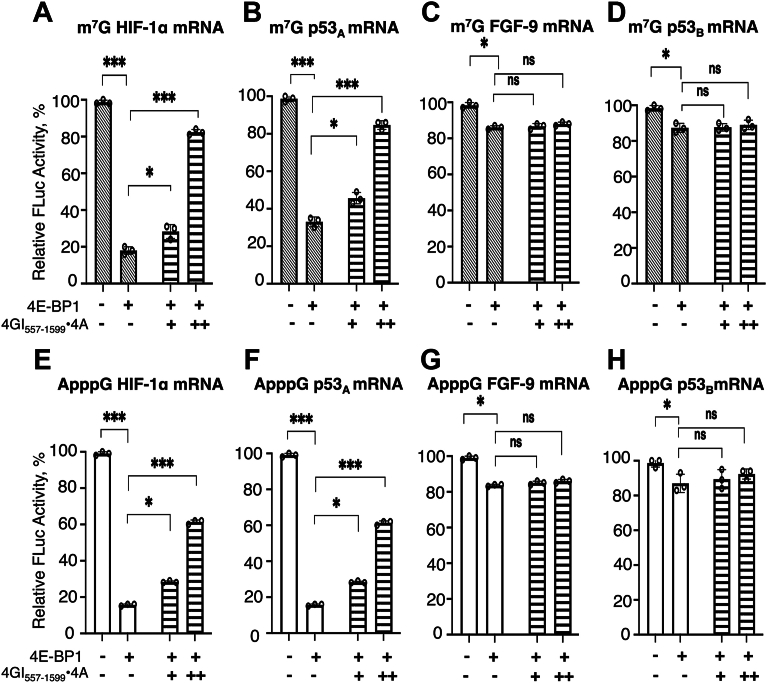
**Effect of eIF4GI_557-1599_**•**eIF4A on the translation yields of m^7^G- and ApppG-capped HIF-1α, p53_A_, FGF-9, and p53_B_-5′UTR-Luc mRNA transcripts**. Translation yields of m^7^G-capped (*A*–*D*) and ApppG-capped (*E*–*H*) mRNA transcripts of HIF-1α-, p53_A_-, FGF-9-, and p53_B_-UTR-Luc mRNAs, respectively, following treatment of the RRL with 50 nM of 4E-BP1 and increasing concentrations of eIF4GI_557-1599_•eIF4A (added at a 1:1 M ratio). Conditions included 50 nM (denoted as +), and 150 nM (denoted as ++) of eIF4GI_557-1599_•eIF4A. For m^7^G-capped mRNAs, 4E-BP1 alone (*diagonally striped bar*, --); increasing eIF4GI•eIF4A concentration (*horizontally striped bar*, --). For ApppG-capped mRNAs, 4E-BP1 alone (*white bar*, --); increasing eIF4GI•eIF4A concentration (*horizontally striped bar*, --). 4E-BP1 was held constant at 50 nM in all conditions. Relative luciferase activity was normalized to the respective controls (m^7^G-capped-UTR-Luc mRNA) for each reporter constructs with no 4E-BP1 added to RRL. Bar heights and error bars correspond to the average and standard deviations, respectively, of three independent luciferase activity measurements. Data were analyzed by two-tailed unpaired Student’s *t* test, where p represents the probability that differences occurred by chance: n.s, p = 0.12; ∗, p < 0.033; ∗∗, p = 0.002; ∗∗∗, p < 0.001.

Given that eIF4GI_557-1599_ can directly bind mRNAs (34), recruit the 43S PIC (34, 43), and promote cap-independent translation of both IRES-like (34, 43, 67) and CITE-like mRNAs (34), its role in overcoming 4E-BP1-mediated translational inhibition was evaluated. Because eIF4A functions as a helicase partner of eIF4GI_557-1599_ within the eIF4F complex, facilitating mRNA remodeling and 43S PIC recruitment (68, 69), both factors were tested in combination (hereafter, denoted as eIF4GI_557-1599_•eIF4A) to assess whether it could restore the 4E-BP1-mediated translation inhibition. To evaluate this, relative luciferase activity was measured from m^7^G-capped UTR-Luc reporter mRNAs in the presence of 50 nM 4E-BP1 and increasing concentrations of eIF4GI_557-1599_•eIF4A (added at a 1:1 M ratio to allow optimal complex formation). For CITE-like HIF-1α-UTR-Luc, and p53_A_-UTR-Luc mRNAs, translation was significantly restored with increasing concentrations of eIF4GI_557-1599_•eIF4A, with relative luciferase activity increasing from ∼20% and ∼36% (at 0 nM added eIF4GI_557-1599_•eIF4A) to ∼82% and ∼85% respectively (at 150 nM added eIF4GI_557-1599_•eIF4A) (Fig. 5, *A* and *B*). A comparable pattern was observed for the ApppG-capped versions of these transcripts, where translation was restored from ∼15% and ∼17% to ∼61% and ∼80%, respectively (Fig. 5, *E* and *F*). These results demonstrated a ∼4-5-fold increase in translation of CITE-like mRNAs, highlighting that eIF4GI_557-1599_, in concert with eIF4A, can overcome 4E-BP1-mediated translation repression and promote cap-independent translation. This is consistent with previous reports showing eIF4GI_557-1599_•eIF4A interaction responsible for 43S PIC recruitment enhances cap-independent translation of viral mRNAs (66, 70, 71).

In contrast, the translation of IRES-like FGF-9-UTR-Luc, and p53_B_-UTR-Luc mRNAs in the presence of 50 nM 4E-BP1 was minimally affected by eIF4GI_557-1599_•eIF4A addition, indicating that these mRNAs may rely on an alternative mode of initiation—potentially scaffold- or helicase-driven—distinct from the eIF4E-dependent pathway. For both m^7^G- and ApppG-capped FGF-9-UTR-Luc, and p53_B_-UTR-Luc mRNAs, the modest inhibition observed with 4E-BP1 was not alleviated by increasing concentrations of eIF4GI_557-1599_•eIF4A (Fig. 5, *C*–*D*, and *G*–*H*). As a control, m^7^G-capped β-actin-UTR-Luc mRNA was used which exhibited modest changes in relative luciferase activity upon eIF4GI_557-1599_•eIF4A addition (Fig. S5), consistent with prior reports (66).

### Complexed with eIF4E•4E-BP1 but increased affinity for ApppG-capped mRNAs.eIF4GI_557-1599_ alone or in complex with eIF4A shows comparable binding affinity for m^7^G-capped mRNAs

While earlier studies have shown that eIF4GI_557-1599_ can directly bind mRNAs (34), the effect of the eIF4E•4E-BP1 on this interaction has not been fully explored. To address this, normalized fluorescence anisotropy assays were performed using m^7^G- or ApppG-capped 5′UTRs derived from HIF-1α, p53_A_, FGF-9, and p53_B_ encoding mRNAs. eIF4GI_557-1599_ binding was assessed both to free mRNA and to mRNA pre-assembled with eIF4E and 4E-BP1 (hereafter referred to as the mRNA•eIF4E•4E-BP1 ternary complex). Anisotropy values were plotted as a function of increasing eIF4GI_557-1599_ concentration, either alone or in complex with eIF4A ± ATP, where ATP was included to test nucleotide-dependent effects on binding.

**Figure 6:**
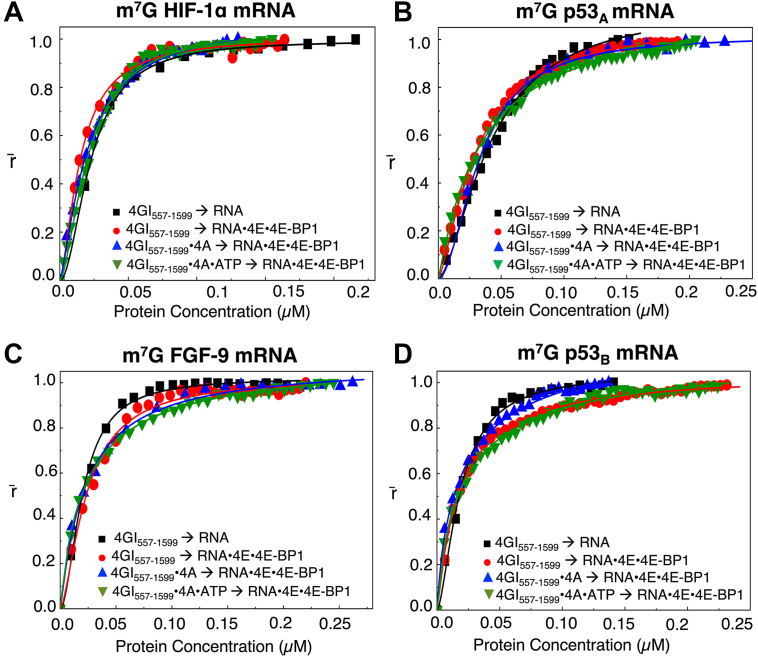
**Fluorescence anisotropy analyses of eIF4GI_557-1599_ (alone or in complex with eIF4A** ± **ATP) binding to m^7^G-capped 5′UTRs of HIF-1α, p53_A_, FGF-9, and p53_B_ encoding mRNAs either alone or as part of the mRNA**•**eIF4E**•**4E-BP1 ternary complex**. Normalized anisotropy changes (denoted as r̄) for the interaction of eIF4GI_557-1599_ binding to mRNAs alone (-▪-) and mRNA•eIF4E•4E-BP1 (); eIF4GI_557-1599_•eIF4A binding to mRNA•eIF4E•4E-BP1 (), and eIF4GI_557-1599_•eIF4A•ATP to mRNA•eIF4E•4E-BP1 () with 10 nM m^7^G-capped 5′UTR of (*A*) HIF-1α, (*B*) p53_A_, (*C*) FGF-9 and (*D*) p53_B_ encoding mRNA transcripts fluorescein-labeled at 3′terminus, respectively. Data points corresponding to the average from three independent anisotropy measurements were normalized and plotted against protein concentration (μ M). Curves represent Hill-equation fits, as described in materials and methods, used to calculate the corresponding K_D_ values.

For m^7^G-capped mRNAs, eIF4GI_557-1599_ exhibited comparable binding affinities across all conditions tested. For the m^7^G-capped HIF-1α 5′UTR mRNA, the K_D_ values were (22 ± 0.5) nM for mRNA alone and (15 ± 3.4) nM when the mRNA was pre-bound by eIF4E•4E-BP1, with minimal changes upon addition of eIF4A or eIF4A•ATP to eIF4GI_557-1599_ (20 ± 1.3, and 21 ± 1.5 nM, respectively). The m^7^G-capped p53_A_ 5′UTR mRNA showed similar behavior, with K_D_ values of (44 ± 3.1) nM (mRNA alone), (31 ± 5.9) nM (mRNA•eIF4E•4E-BP1 bound), and minor changes with eIF4A or eIF4A•ATP (33 ± 0.7, and 35 ± 1.3 nM, respectively). (Table 1, Fig. 6, *A* and *B*). For the m^7^G-capped FGF-9 5′UTR mRNA, K_D_ values were (20 ± 2.0) nM (mRNA alone) and (24 ± 0.9) nM (mRNA•eIF4E•4E-BP1 bound), with negligible effects from eIF4A or eIF4A ATP (23 ± 1.3, and 24 ± 1.5 nM, respectively). Similarly, the m^7^G-capped p53_B_ 5′UTR mRNA showed K_D_ values of (18 ± 3.1) nM (mRNA alone), (18 ± 0.3) nM (mRNA•eIF4E•4E-BP1 bound), and (21 ± 2.2, and 22 ± 1.1 nM, respectively) with eIF4A or eIF4A•ATP (Table 1, Fig. 6, *C* and *D*). These results likely suggest that for m^7^G-capped mRNAs, eIF4GI_557-1599_ can bind directly to the mRNA at sites distinct from the cap-bound eIF4E•4E-BP1 complex, resulting in largely unchanged affinities

**Figure 7:**
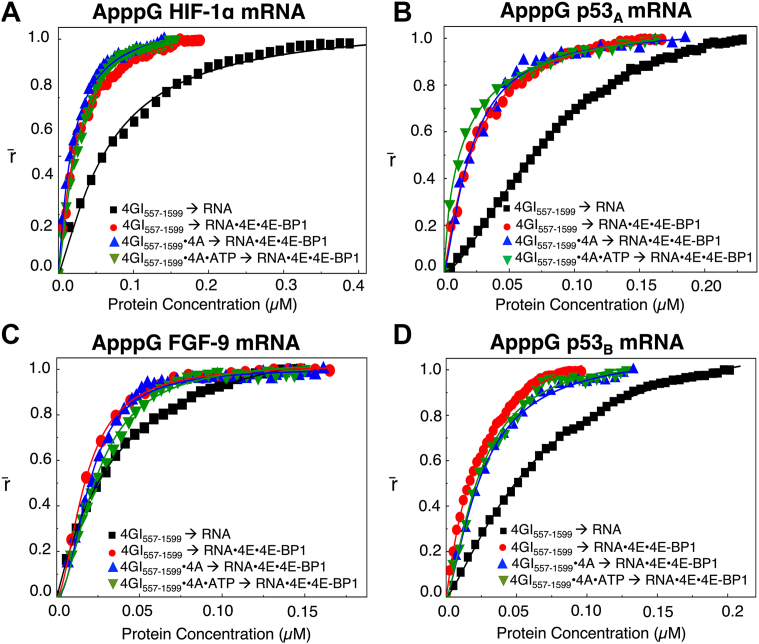
**Fluorescence anisotropy analyses of eIF4GI_557-1599_ (alone or in complex with eIF4A** ± **ATP) binding to ApppG-capped 5′UTRs of HIF-1α, p53_A_, FGF-9, and p53_B_ encoding mRNAs either alone or as part of the mRNA**•**eIF4E**•**4E-BP1 ternary complex**. Normalized anisotropy changes (denoted as r̄) for the interaction of eIF4GI_557-1599_ binding to mRNAs alone (-▪-) and mRNA•eIF4E•4E-BP1 (); eIF4GI_557-1599_•eIF4A binding to mRNA•eIF4E•4E-BP1 (), and eIF4GI_557-1599_•eIF4A•ATP to mRNA•eIF4E•4E-BP1 () with 10 nM ApppG-capped 5′UTR of (*A*) HIF-1α, (*B*) p53_A_, (*C*) FGF-9 and (*D*) p53_B_ encoding mRNA transcripts fluorescein-labeled at 3′terminus, respectively. Data points corresponding to the average from three independent anisotropy measurements were normalized and plotted against protein concentration (μ M). Curves represent Hill-equation fits, as described in materials and methods, used to calculate the corresponding K_D_ values.

Unlike m^7^G-capped mRNAs, where eIF4GI_557-1599_ binding remained largely unchanged, ApppG-capped mRNAs exhibited enhanced binding. For the ApppG-capped 5′UTR of HIF-1α mRNA, the K_D_ values decreased from (67 ± 1.8) nM for mRNA alone to (25 ± 1.8) nM when mRNA was pre-bound by the eIF4E•4E-BP1 and was (19 ± 0.6) nM and (24 ± 4.3) nM when eIF4GI_557-1599_ was complexed with eIF4A or eIF4A•ATP, respectively. Likewise, for the ApppG-capped p53_A_ 5′UTR mRNA, the K_D_ values decreased from (81 ± 1.3) nM to (25 ± 1.1) nM and was (20 ± 2.0) nM and (16 ± 0.8) nM when eIF4GI_557-1599_ was complexed with eIF4A or eIF4A•ATP, respectively (Table 2, Fig. 7, *A* and *B*). These results indicated a ∼3-4-fold increase in eIF4GI_557-1599_ binding affinity upon mRNA•eIF4E•4E-BP1 ternary complex formation. Enhanced eIF4GI_557-1599_ binding was also evident for the ApppG-capped 5′UTRs of FGF-9, and p53_B_ mRNAs. The K_D_ values for the 5′UTR of FGF-9 mRNA decreased from (28 ± 0.7) nM for mRNA alone to (13 ± 0.6) nM in the presence of the eIF4E•4E-BP1 and was (15 ± 1.0) nM and (18 ± 1.0) nM when eIF4GI_557-1599_ was complexed with eIF4A or eIF4A•ATP, respectively (Table 2, Fig. 7*C*). Similarly, for the ApppG-capped 5′UTR of p53_B_ mRNA, the K_D_ values decreased from (66 ± 1.7) nM, to (22 ± 0.8) nM and was (28 ± 1.8) nM and (21 ± 1.1) nM when eIF4GI_557-1599_ was complexed with eIF4A or eIF4A•ATP, respectively (Table 2, Fig. 7*D*), representing a ∼2-fold increase in binding affinity. This pattern likely suggests that for ApppG-capped mRNAs, both eIF4E•4E-BP1 and eIF4GI_557-1599_ can interact with mRNA simultaneously, resulting in tighter binding. Hence, for both m^7^G- and ApppG-capped mRNAs, eIF4GI_557-1599_ retains the ability to bind directly to the mRNA.

### eIF4GI_557-1599_ does not bind the eIF4E•4E-BP1 complex, suggesting mRNA-mediated recruitment

It was expected that eIF4GI_557-1599_ would not bind to the eIF4E•4E-BP1 complex because 4E-BP1 blocks the eIF4GI_557-1599_ binding site. To test this assumption, fluorescence anisotropy assays were performed with a fluorescein-labeled 4E-BP1 peptide (residues PGGTRIIYDRKFLMECRNSP, with Fluorescein isothiocyanate (FITC) conjugated at the Lys residue) in the absence of mRNA, thereby distinguishing direct protein–protein interactions from mRNA-mediated binding. This 20-residue sequence includes the canonical eIF4E-binding motif and has been widely used to recapitulate the binding affinity of full-length 4E-BP1 in biochemical studies (47, 72). Titration of eIF4E into FITC–4E-BP1 peptide resulted in a marked increase in anisotropy with a K_D_ value of (5 ± 3.7) nM (Fig. S6), consistent with the high-affinity binding between eIF4E and the 4E-BP1 peptide (73). Note that, due to detection limits, there is a huge degree of uncertainty in the K_D_ values below 10 nM. In contrast, titration of eIF4GI_557-1599_ into either the free FITC–4E-BP1 peptide or the pre-formed FITC–4E-BP1•eIF4E showed negligible changes in anisotropy (Fig. S6). These results indicated that eIF4GI_557-1599_ does not bind to the 4E-BP1 peptide or eIF4E•4E-BP1 in the absence of mRNA (Fig. S6), indicating that any eIF4GI_557-1599_ binding observed in mRNA-based assays (Tables 1 and 2, Fig. 6, *A*–*D*, and 7, *A*–*D*) reflects mRNA-dependent interactions rather than direct protein-protein interaction with eIF4E•4E-BP1. eIF4GI_557-1599_ likely binds directly with internal or structural elements within the 5′UTRs, potentially stabilized in the mRNA•eIF4E•4E-BP1 complex, aligning with previous reports that direct eIF4GI_557-1599_-mRNA interactions facilitate translation under conditions where cap-dependent translation is impaired (38).

### eIF4GI_557-1599_ cannot displace the FITC-labeled 4E-BP1 peptide from the eIF4E–mRNA complex

**Figure 8:**
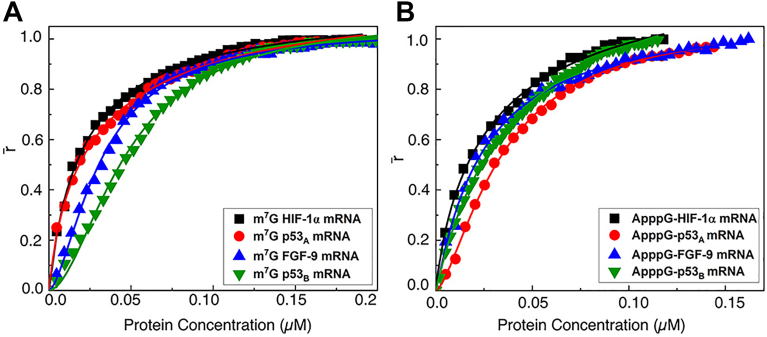
**Fluorescence anisotropy analyses of eIF4GI_557-1599_ binding to mRNA•eIF4E•4E-BP1-FITC-Peptide ternary complex**. Normalized anisotropy changes (denoted as r̄) for the interaction of eIF4GI_557-1599_ binding to pre-assembled mRNA•eIF4E•4E-BP1-FITC-peptide ternary complexes in the presence of different 5′capped mRNAs. Each mRNA was tested in both m^7^G-capped (*A*) and ApppG-capped (*B*) forms. 5′UTRs of mRNA encoding HIF-1α (-▪-), p53_A_ (), FGF-9 (), and p53_B_ () were analyzed. All assays were performed using 20 nM fluorescein-labeled 4E-BP1 peptide. Data points corresponding to the average from three independent anisotropy measurements were normalized and plotted as a function of eIF4GI_557-1599_ concentration (μ M). Curves represent Hill-equation fits, as deciphered in materials and methods, used to calculate the corresponding K_D_ values.

To examine the extent to which eIF4GI_557-1599_ can displace 4E-BP1 from the eIF4E•4E-BP1, fluorescence anisotropy assays were performed using a FITC-labeled 4E-BP1 peptide pre-bound to eIF4E in the presence of various capped mRNAs. Increasing concentrations of eIF4GI_557-1599_ were titrated into pre-formed eIF4E•4E-BP1-FITC-peptide containing either m^7^G-capped or ApppG-capped 5′UTRs of HIF-1α, p53_A_, FGF-9, or p53_B_ encoding mRNAs. Displacement of the labeled 4E-BP1 peptide by eIF4GI_557-1599_ would be expected to result in a decrease in anisotropy due to 4E-BP1 peptide dissociation, whereas simultaneous binding would be expected to increase anisotropy. Across all tested mRNAs, anisotropy consistently increased with eIF4GI_557-1599_ titration, indicating that eIF4GI_557-1599_ does not displace 4E-BP1 from eIF4E•4E-BP1, and that the observed binding in mRNA-based assays possibly arises from eIF4GI_557-1599_ interacting directly with mRNA rather than competing for the eIF4E-binding site. Importantly, this effect was independent of the cap structure, as similar K_D_ values were observed for m^7^G- and ApppG-capped mRNAs. For example, K_D_ values for eIF4GI_557-1599_ binding were (21 ± 0.7) nM vs. (30 ± 3.1) nM for the 5′UTR of HIF-1α, (29 ± 1.3) nM vs. (35 ± 1.5) nM for the 5′UTR of p53_A_, (32 ± 0.3) nM vs. (25 ± 1.0) nM for the 5′UTR of FGF-9, (47 ± 0.7) nM vs. (42 ± 0.7) nM for the 5′UTR of p53_B_ mRNAs, respectively (Tables 1 and 2, Fig. 8, *A* and *B*), comparing m^7^G- and ApppG-capped mRNA transcripts. Thus, eIF4GI_557-1599_ binding in these assays reflects direct interaction with the mRNA, potentially stabilized by structural features or conformational changes in the mRNA•eIF4E•4E-BP1 complex rather than displacement of 4E-BP1.

## Discussion

In this study, fluorescence-based steady state anisotropy assays and *in-vitro* translation assays were used to explore the quantitative binding and mechanisms by which elevated 4E-BP1 levels influence eIF4E-independent translation of a subset of cellular mRNAs. Building on a previous finding that 5′end accessibility is essential for 43S PIC recruitment in CITE-like translation of HIF-1α-UTR-Luc (16), and p53_A_-UTR-Luc mRNAs, but dispensable for IRES-like FGF-9-UTR-Luc, or p53_B_-UTR-Luc mRNAs (16, 34)—this study examined how 4E-BP1 modulates these distinct mechanisms and can have a differential effect on the translation of each subset of mRNAs.

It was observed that the eIF4E•4E-BP1 bound both m^7^G-capped and ApppG-capped CITE-like (*e*.*g*., HIF-1α-UTR and p53_A_-UTR) mRNAs with ∼4-5-fold higher binding affinities than eIF4E alone (Fig. 1, *A* and *B*, and 2, *A* and *B*), consistent with reports that eIF4E binds mRNA *via* enthalpy-driven interactions (74), further stabilized by the conserved PGVTS/T motif of 4E-BP1 enhancing binding ∼1000-fold compared to eIF4E binding to cap analogs (51, 74, 75). Structural features such as stem-loops in CITE-like mRNAs (43) may contribute to this regulatory mechanism, particularly under stress, where stabilized eIF4E•4E-BP1 may shift translation from cap-dependent to eIF4E-independent, selectively promoting translation of mRNAs crucial for cellular adaptation and survival. The observed transcript-specific modulation—where eIF4E•4E-BP1 exhibits stronger binding to CITE-like than IRES-like mRNAs—suggests that 4E-BP1 selectively represses or permits translation based on 5′UTR architecture (16).

*In vitro* translation assays showed that elevated 4E-BP1 significantly inhibited the translation of CITE-like (HIF-1α, and p53_A_-UTR-Luc) mRNA transcripts, while minimally affecting IRES-like (FGF-9, and p53_B_-UTR-Luc) mRNAs (Fig. 3, *C*–*F*) although both CITE- and IRES-like mRNA constructs bound eIF4E•4E-BP1 with high affinity. This inhibition corelated with a ∼4-5-fold higher binding affinity of eIF4E•4E-BP1 compared to eIF4E alone for CITE-like mRNAs, likely reflecting restricted 43S PIC access to the 5′end. The effect observed for both functional m^7^G- and non-functional ApppG-capped transcripts indicates that the underlying mechanism depends primarily on 5′UTR structure rather than cap identity.

In contrast, the modest (∼2-fold) increase in eIF4E•4E-BP1 binding to IRES-like mRNAs had little impact on their translation, even at high 4E-BP1 levels (Fig. 3, *E* and *F*). These results are consistent with earlier observations that 5′end hairpins do not impair translation of ApppG-capped FGF-9-UTR-Luc, and p53_B_-UTR-Luc mRNAs (34). This supports a model in which these mRNAs recruit ribosomes internally, independent of 5′end accessibility or eIF4E binding (38, 57), resembling viral and cellular IRES-containing mRNAs that translate independently of the 5′cap (1, 76, 77, 78). The modest increase in eIF4E•4E-BP1 binding likely reflects the distal binding of eIF4GI_557-1599_ or the 43S PIC from the 5′cap (34), making tighter eIF4E•4E-BP1 binding irrelevant. Consequently, these mRNAs are efficiently translated in the presence of 4E-BP1 through direct ribosome recruitment by eIF4GI_557-1599_ (79)—a strategy commonly exploited by viruses to evade 4E-BP1-mediated translational inhibition (80). The efficiency of this mechanism can vary with cellular stress, 4E-BP1 phosphorylation, and eIF availability (81, 82, 83). Overall, IRES-like mRNAs promote translation without 5′end scanning, facilitating ribosomal recruitment despite 4E-BP1 inhibition (55, 57, 65, 84). In this context, 4E-BP1 has been shown to drive translational reprogramming under stress, promoting cap-independent translation (44, 85, 86). Collectively, these findings suggest that 5′UTR architecture, rather than cap identity, dictates mRNA sensitivity to 4E-BP1. This observation raises the possibility that transcripts such as HIF-1α—previously classified as IRES-driven (94)—may instead function through a CITE-like mechanism (36, 95), underscoring the need to refine mRNA translational classification based on structural and functional features.

To examine adaptive translation under elevated 4E-BP1, we assessed the effects of eIF4E and eIF4GI_557–1599_•eIF4A on CITE-like and IRES-like mRNAs. Under elevated 4E-BP1, eIF4GI_557–1599_•eIF4A effectively alleviated much of the translational repression of CITE-like mRNAs, but not the modest inhibition of IRES-like ones (Fig. 5, *A*–*H*). This suggests that recruitment of the eIF4GI_557–1599_ scaffold with eIF4A helicase—can substitute for impaired eIF4E function by promoting 43S PIC loading near the 5′end. Consistent with this, eIF4GI_557–1599_ alone also exhibited a similar trend toward restoring CITE-like mRNA translation (data not included). Such eIF4GI_557–1599_-dependent, eIF4E-independent initiation has been reported for several stress-responsive mRNAs, including 53BP1, HIF-1α, p53, BRCA1, and GADD45a (87). Elevated eIF4GI_557-1599_ levels may enhance ribosome–mRNA interactions or stabilize initiation at the 5′end (88, 89, 90), thereby reducing cap dependency and conferring resistance to 4E-BP1-mediated inhibition (38, 91, 92).

Increasing eIF4E levels under high 4E-BP1 conditions also partially restored CITE-like mRNA translation, likely by re-establishing functional eIF4F complexes that outcompete 4E-BP1 for eIF4E binding. In contrast, addition of eIF4E had modest effect on IRES-like mRNAs (Fig. 4, *A*–*H*), underscoring their differential reliance on cap-dependent initiation. This agrees with studies indicating that eIF4E promotes translation of highly structured, growth-promoting mRNAs linked to cellular transformation and tumor progression (93, 94).

**Figure 9:**
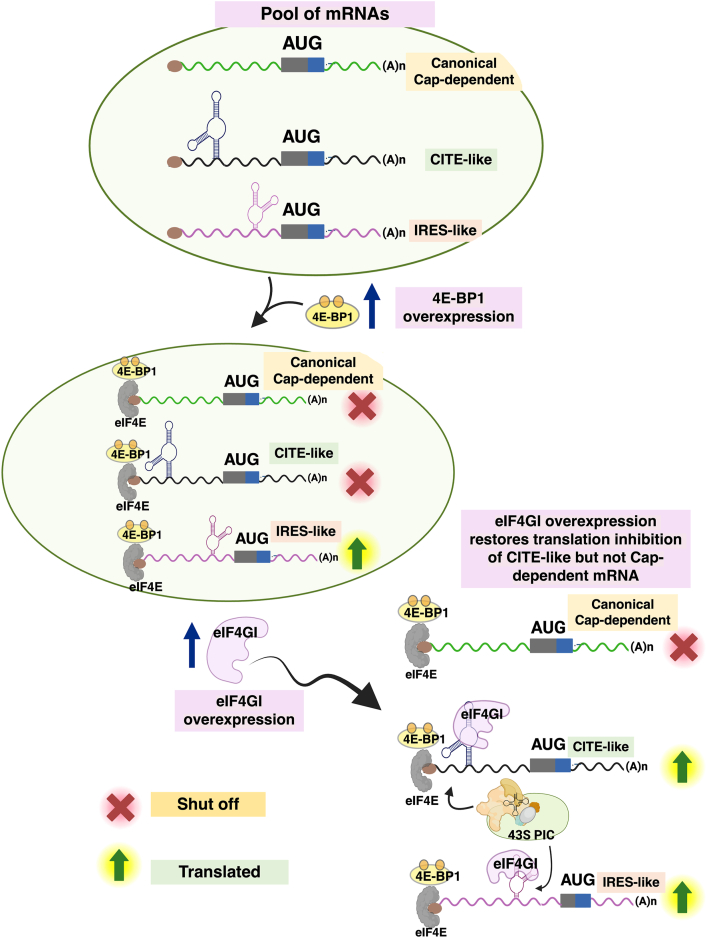
**Proposed model illustrating the differential effects of 4E-BP1 and eIF4GI_557-1599_ on cap-independent translation of CITE-like and IRES-like mRNAs**. Under normal conditions, a pool of mRNAs—including canonical cap-dependent, CITE-like, and IRES-like transcripts—is available for translation. Overexpression of 4E-BP1, such as under stress conditions, promotes sequestration of eIF4E. The resulting eIF4E•4E-BP1 binds to the m^7^G-cap of these mRNAs, blocking 43S PIC recruitment at the 5′ m^7^G-cap and inhibiting translation of canonical cap-dependent and CITE-like mRNAs, while IRES-like mRNAs remain minimally affected. Subsequent overexpression of eIF4GI_557-1599_ enhances its availability to interact with mRNAs. eIF4GI_557-1599_ restores translation inhibition of CITE-like mRNAs, likely by directly interacting with the structured 5′UTRs but fails to rescue translation of cap-dependent mRNAs and has limited impact on IRES-like mRNAs whose translation is inherently resistant to 4E-BP1-mediated translation repression. CITE, cap-independent translation enhancer; eIF, eukaryotic initiation factor; IRES, internal ribosome entry site; m^7^G, methyl guanosine; PIC, preinitiation complex.

Overall, these findings lead to the model illustrated in Figure 9: eIF4E•4E-BP1 binds tightly to canonical cap-dependent mRNA and inhibits translation by preventing preinitiation complex recruitment. CITE-like mRNAs are similarly inhibited by eIF4E•4E-BP1; however, this inhibition can be alleviated through overexpression of eIF4GI_557-1599_, which restores translation—unlike in the canonical cap-dependent context. IRES-like mRNAs, while binding eIF4E•4E-BP1 relatively tightly, are minimally affected by eIF4E•4E-BP1. Taken together, these findings suggest a synergistic interaction of 4E-BP1 and eIF4GI_557-1599_ in selective modulation of protein synthesis. This model highlights how 4E-BP1 and eIF4GI_557-1599_ differentially regulate both cap-dependent and cap-independent translation of CITE-like and IRES-like mRNAs. Such selective modulation of mRNA translation under stress conditions lays the groundwork for therapeutic approaches targeting translation in stress-related diseases.

## Experimental procedures

### Purification of recombinants eIF4GI_557-1599_, eIF4E, 4E-BP1, 4E-BP1 mutant (4E-BP1-4Ala), and eIF4A

The plasmids encoding human eIF4GI_557-1599_ with an N-terminal 6x-histidine tag and a TEV protease cleavage site was a generous gift from Dr Christopher S. Fraser. The human 4E-BP1 coding sequence (GenBank: BC058073.1) was obtained from the NCBI protein database. A dominant-negative mutant, 4E-BP1-4Ala (T37 A, T46 A, S65 A, T70 A), was designed by mutating key phosphorylation sites to alanine (61). The corresponding nucleotide sequences of wild-type 4E-BP1 and 4E-BP1-4Ala were subcloned into the NdeI and XhoI sites of a modified pET28a-TEV expression vector, incorporating an N-terminal 6 × histidine tag. The wild-type 4E-BP1 plasmid was obtained from GenScript, while the 4E-BP1-4Ala plasmid was purchased from AddGene. Plasmid encoding eIF4GI_557-1599_, eIF4E, 4E-BP1 and 4E-BP1-4Ala were transformed into *Escherichia coli* BL21 (DE3) competent cells following the New England Biolabs transformation protocol for protein expression. Figure S7 illustrates the domain organization of the proteins analyzed in this study.

All these proteins were recombinantly expressed in E.coli BL21CodonPlus (DE3)-RIL cells (Agilent). 4E-BP1 and 4E-BP1-4Ala was purified from bacterial cell lysates using Ni^2+^-nitrilotri-acetic acid (Ni-NTA) affinity (GE HealthCare Life Sciences) and HisPur Ni-NTA spin column (Thermo Fisher Scientific), as per the manufacturer’s instructions (34). eIF4GI_557-1599_, was purified using a combination of nickel-nitrilotriacetic acid (Ni-NTA) affinity followed by heparin affinity columns, as previously described (34). The eIF4A clone contained an N-terminal His–MBP fusion tag followed by a TEV protease cleavage site. eIF4A was expressed in *E*. *coli* BL21 (DE3) cells and purified by Ni–NTA affinity chromatography, followed by TEV cleavage and buffer exchange, as described previously (24). The purified 6x-histidine tagged proteins were dialyzed overnight against storage buffer (20 mM HEPES–KOH pH 7.6, 200 mM KCl, 10 mM β-mercaptoethanol, and 10% glycerol) in the presence of tobacco etch virus (TEV) protease to cleave off the tags (95). The untagged proteins were further purified and concentrated using 1 ml HiTrap Heparin HP columns (GE HealthCare Life Sciences) (34). The eluted proteins were analyzed on 10% SDS-PAGE gels, and pure fractions (>95% purity) were pooled and dialyzed overnight against storage buffer. For visual confirmation and analysis of protein purity, samples were run on SDS-PAGE and stained with Coomassie Brilliant Blue (Thermo Fisher Scientific). (34, 74). Aliquots were then stored at −80 °C. Proteins were then concentrated, and their concentrations were quantified using Bradford assay (standard curve method). 4E-BP1, and 4E-BP1-4Ala were analyzed using a 10% SDS-PAGE gel.

The human eIF4E clone was also a kind gift from Prof. Christopher S. Fraser (UC Davis). This eIF4E construct had an N-terminal polyhistidine tag, with a TEV protease cleavage site between the histidine tag and eIF4E. For expression of recombinant eIF4E protein, *E*. *coli* Rosetta (DE3) was transformed with this clone and bacterial cells from 1.5 L of LB medium were used. The bacterial cells were grown at 37°C till the A_600_ reached 0.6 to 0.8. Protein expression was induced overnight at 20°C, by adding IPTG (final concentration of 0.5 mM). The cells were pelleted and sonicated in the lysis buffer (25 mM HEPES, pH 7.5, 300 mM KCl, 10% glycerol, 1 mM DTT, 20 mM imidazole, and protease inhibitor tablet). The supernatant was filtered through a 400-micron syringe filter and loaded on a pre-equilibrated 5 ml Ni-NTA column (pre-equilibrated with the lysis buffer). The column was washed with 20 ml buffer E (25 mM HEPES, pH 7.5, 300 mM KCl, 10% glycerol, 1 mM DTT, and 50 mM imidazole), and the protein was eluted with buffer E containing 500 mM imidazole. Fractions containing eIF4E were pooled and dialyzed overnight against buffer E (without any imidazole) containing TEV protease, at 4 °C. The dialyzed samples were further purified on a 5 ml Q-Sepharose column to separate the untagged protein from the cleaved tag and the TEV protease. The KCl concentration of dialyzed samples was adjusted to 100 mM, and the sample was loaded onto a 5 ml preequilibrated Q-Sepharose column (preequilibrated with buffer A 25 mM HEPES pH 7.5, 100 mM KCl, 10% glycerol, and 1 mM DTT). The proteins were eluted using a step gradient of 150 to 500 mM KCl in buffer A. Fractions containing eIF4E, were pooled, concentrated, and stored at −80 °C. The protein concentrations were quantified using the Bradford’s assay and purity was assessed by 10% SDS-PAGE gel (Fig. S8).

### Preparation of mRNAs for fluorescence anisotropy-based equilibrium binding studies

DNA templates corresponding to the 5′UTRs of mRNAs encoding HIF-1α (294 nucleotides [nt]; GenBank accession no. AH006957.2), FGF-9 (177 nt; GenBank accession no. AY682094.1), one isoform of p53 (p53_A_) (136 nt; GenBank accession no. JN900492.1), the 5′UTR for a second isoform of p53 (p53_B_) (117 nt; GenBank accession no. MG595994.1), and β-actin (84 nt; GenBank accession no. AK301372.1), respectively (Table S1) were purchased from Integrated DNA Technologies, and the corresponding mRNAs were synthesized *via in-vitro* transcription using the HiScribe T7 Quick High Yield RNA Synthesis Kit (New England Biolabs Inc) following the manufacturer’s protocol (34, 74). The purified mRNAs were then labeled at the 3′ termini in an oxidation reaction using a final concentration of 30 mM mRNA,105 mM of NaOAc pH-5.2, and 15 mM of sodium periodate. The reaction tube was covered with aluminum foil and incubated for 30 min in the dark (34, 74). After incubation, 1 M sodium sulfite was added and incubated for an additional 10 min in the dark. The oxidation product was purified using ethanol precipitation. The purified oxidized mRNA was then used for a labeling reaction by adding a final concentration of 1 mM flouroscein-5-thiosemicarbazide (FITC) and 50 mM Na-phosphate buffer pH 6.5 (34). The reaction was covered with aluminum foil and incubated in the dark for 2 h. Following this incubation period, 1 M NaCNBH_3_ was added, and the reaction was further incubated overnight at 4 °C. After overnight incubation, the labeled product was purified using a two-step purification. Ethanol precipitation followed using the mRNA Clean and Concentrator Kit from Zymo Research following the manufacturer’s protocol (34). The purified and labeled mRNAs were then capped with Ribo m^7^G Cap Analog (Promega), catalog number P1802, using the vaccinia capping system, catalog number M2080S from New England Biolabs. For the ApppG capped analog, G(5′)ppp(5′)A RNA Cap structure Analog (New England Biolabs Inc) was used. These RNAs were synthesized using Co-transcriptional capping using the HiScribe T7 Quick High Yield RNA Synthesis Kit. Briefly, 5′end of the mRNAs was capped, 3′termini were labeled with FITC, and purified following previously discussed protocol (34). The mRNA concentrations were determined using nano-drop UV-visible spectrometer and integrity was verified by 1.5% agarose gel electrophoresis (34).

### Binding assay using fluorescence anisotropy

Fluorescein isothiocyanate (FITC) (Sigma Aldrich) labeled mRNAs (FGF-9, HIF-1α, p53_A_, p53_B_) were diluted to 10 nM in binding buffer (20 mM HEPES-KOH, pH 7.5, 200 mM KCl, and 1 mM MgCl_2_). Fluorescence anisotropy experiments were performed to assess the binding of 3′ labeled 5′UTR of mRNAs encoding FGF-9, HIF-1α, and two isoforms of p53 (p53_A_ and p53_B_) with eIF4E, eIF4E in absence and presence of 4E-BP1, and 4E-BP1 alone. Binding experiments were carried out using the equilibrium titration module of an SF-300X stopped-flow fluorimeter (KinTek Corporation) and a temperature controller (Fisher Scientific). Fluorescein-labeled RNAs were excited at 495 nm and resulting emission was detected at 515 nm using blocking edge BrightLine long pass filter (Semrock Inc.). For each assay, 200 μl of 10 nM of fluorescein-labeled mRNA was loaded into the cuvette and titrated by automated continuous injection of 20 μl of varying concentrations of proteins or protein complexes over a period of 20 min at 25 °C.

To assess mRNA binding, 10 nM mRNA was incubated either alone or after preassembly with 0.5 μM eIF4E and 1 μM 4E-BP1 (1:2) for 20 min. Titrations were then performed with 2.5 μM eIF4GI_557-1599_ (alone or in complex with eIF4A/ATP). To assess peptide binding, a FITC-labeled 4E-BP1 peptide (sequence: PGGTRIIY[K(FITC)]DRFLMECRNSP; ≥98% purity; GenScript) was used at 20 nM. The peptide was tested either free in solution or after preincubation with 10 nM eIF4E for 20 min at 25 °C. Where indicated, 4E-BP1-peptide•eIF4E complex was further incubated with 10 nM mRNA to form a ternary mRNA•eIF4E•4E-BP1-peptide complex prior to titration with eIF4GI_557-1599_. Direct eIF4E–4E-BP1 peptide interactions were measured by titrating 200 μl of 20 nM peptide with 2.5 μM eIF4E. 50 data points were collected for each anisotropy measurement. Data from three independent binding assays were averaged and used to calculate dissociation equilibrium constants (K_D_). The K_D_ values were calculated by fitting the Hill equation using Origin Pro8 software (https://www.originlab.com/demodownload.aspx),

$$r_{\text{obs}}=r_{\min}+\left(r_{\max}-r_{\min}\right)\left[\frac{\left[\text{Protein}\right]}{K_{D}+\left[\text{Protein}\right]}\right]$$

where r_obs_ is the observed anisotropy value, r_min_ is the minimum anisotropy value in the absence of protein (4E-BP1), r_max_ is the final saturated anisotropy value, [Protein] is the concentration of eIF4E•4E-BP1 complex and K_D_ is the equilibrium dissociation constant. The chi-squared values (χ^2^) that represented the statistical goodness of fit were always close to 1 (Tables 1 and 2). Fitting data to a two-site model did not improve the fit as judged by (χ^2^) values.

### Preparation of the UTR-Luc reporter mRNAs for *in-vitro* translation assays

The UTR-Luciferase mRNA constructs for the luciferase gene expression reporter assay were generated from the glycerol stocks gifted by Dr Solomon Haizel. The plasmids were cultured with ampicillin, purified with QIAprep Spin miniprep kit (Qiagen) following the manufacturer’s protocol, then linearized using Acc65I (ThermoScientific) and purified using the GeneJET gel extraction and DNA cleanup micro kit per manufacturer’s instructions (34). DNA templates were *in -vitro* transcribed using a T7 RiboMax large-scale RNA production kit (Promega) following the manufacturer’s protocol. ApppG (NEB) or Ribo m^7^GpppA cap analog (Promega) were added to the transcription mix in an ApppG or Ribo m^7^GpppA:GTP ratio of 10:1 to get mRNA transcripts with non-functional and functional caps, respectively. Capped mRNAs were poly(A) tailed using the poly(A) tailing kit (Invitrogen) following the manufacturer’s protocol. The resulting capped and polyadenylated mRNAs were then purified using a Monarch RNA Cleanup kit, 50 μg (*New England Biolabs*) following the manufacturer’s protocol. RNA concentrations were determined using a Nano-drop UV-visible spectrometer.

### Luciferase-based gene expression reporter assays

*In vitro* translation assays were performed by adding 1 μg of UTR-Luc mRNAs to 25 μl *in-vitro* reaction mixtures made using the Rabbit Reticulocyte Lysate (RRL) *in-vitro* translation system from Promega. The reaction mixtures contained 70% (v/v) RRL (Promega), 1 mM amino acid mixture (Promega), 75 mM KCl (Fisher Chemical), 0.5 mM MgCl_2_ (Invitrogen) and 10 units/μl RiboLock RNase inhibitor (ThermoScientific) (24, 34). First, proteins were incubated with lysate mixture for 10 min at 30 °C in the absence of mRNA (64, 65). Then 1 μg (60 nM) of UTR-Luc mRNA was added to the RRL mixtures, incubated at 30 °C for 40 min and the reaction was stopped by placing the tubes in ice for 10 min 5 μl of each reaction mixture was added to 40 μl Bright-Glo luciferase assay reagent (Promega) and measured for luminescence using a SpectraMax iD5 microplate reader (Molecular Devices) with default endpoint luminescence settings. Each reaction was measured in triplicate using 3 different batches of RRLs and analyzed using GraphPad Prism 8. Statistical significance between the mean values were analyzed using two-tailed unpaired student’s *t* test (GraphPad Prism 8 software) (https://www.graphpad.com/). The statistical significance was set at *p* < 0.05 and the *p*-values were calculated. The calculated *p*-values for the analyses are indicated as follows on the brackets above the bar graphs (Figs. n.s (non-significant), *p* = 0.12; ∗, *p* = 0.033; ∗∗, *p* = 0.002; ∗∗∗, *p* < 0.001).

## Data availability

All data are contained in the manuscript. This manuscript contains supporting information.

## Supporting information

This article contains supporting information.

## Conflict of interest

These authors declare they have no conflict of interest with the contents of this article.

## References

[1] Smyth J.W., Shaw R.M. (2013). Autoregulation of connexin43 gap junction formation by internally translated isoforms. Cell Rep..

[2] Raught B.G.A. (1999). eIF4E activity is regulated at multiple levels. Int. J. Biochem. Cell Biol..

[3] Amorim I.S., Lach G., Gkogkas C.G. (2018). The role of the eukaryotic translation initiation factor 4E (eIF4E) in neuropsychiatric disorders. Front. Genet..

[4] Elia A., Constantinou C., Clemens M.J. (2008). Effects of protein phosphorylation on ubiquitination and stability of the translational inhibitor protein 4E-BP1. Oncogene.

[5] Lin T.A., Kong X., Saltiel A.R., Blackshear P.J., Lawrence J.C. (1995). Control of PHAS-I by insulin in 3T3-L1 adipocytes. Synthesis, degradation, and phosphorylation by a rapamycin-sensitive and mitogen-activated protein kinase-independent pathway. J. Biol. Chem..

[6] Zhang D., Contu R., Latronico M.V., Zhang J., Rizzi R., Catalucci D. (2010). mTORC1 regulates cardiac function and myocyte survival through 4E-BP1 inhibition in mice. J. Clin. Invest..

[7] Wang Z., Feng X., Molinolo A.A., Martin D., Vitale-Cross L., Nohata N. (2019). 4E-BP1 is a tumor suppressor protein reactivated by mTOR inhibition in head and neck cancer. Cancer Res..

[8] Tsai S., Sitzmann J.M., Dastidar S.G., Rodriguez A.A., Vu S.L., McDonald C.E. (2015). Muscle-specific 4E-BP1 signaling activation improves metabolic parameters during aging and obesity. J. Clin. Invest..

[9] Bohm R., Imseng S., Jakob R.P., Hall M.N., Maier T., Hiller S. (2021). The dynamic mechanism of 4E-BP1 recognition and phosphorylation by mTORC1. Mol. Cell..

[10] Sonenberg N., Hinnebusch A.G. (2009). Regulation of translation initiation in eukaryotes: mechanisms and biological targets. Cell.

[11] Thakor N., Holcik M. (2012). IRES-mediated translation of cellular messenger RNA operates in eIF2alpha- independent manner during stress. Nucleic Acids Res..

[12] Spriggs K.A., Bushell M., Mitchell S.A., Willis A.E. (2005). Internal ribosome entry segment-mediated translation during apoptosis: the role of IRES-trans-acting factors. Cell Death Differ..

[13] Komar A.A., Hatzoglou M. (2011). Cellular IRES-mediated translation: the war of ITAFs in pathophysiological states. Cell Cycle.

[14] Spriggs K.A., Bushell M., Willis A.E. (2010). Translational regulation of gene expression during conditions of cell stress. Mol. Cell.

[15] Sun R., Cheng E., Velasquez C., Chang Y., Moore P.S. (2019). Mitosis-related phosphorylation of the eukaryotic translation suppressor 4E-BP1 and its interaction with eukaryotic translation initiation factor 4E (eIF4E). J. Biol. Chem..

[16] Shatsky I.N., Terenin I.M., Smirnova V.V., Andreev D.E. (2018). Cap-independent translation: what's in a name?. Trends Biochem. Sci..

[17] Ramirez-Valle F., Braunstein S., Zavadil J., Formenti S.C., Schneider R.J. (2008). eIF4GI links nutrient sensing by mTOR to cell proliferation and inhibition of autophagy. J. Cell Biol..

[18] Ozretić P.B., Alessandra & Inga A., Levanat S. (2012). The growing relevance of cap-independent translation initiation in cancer-related genes. Periodicum Biologorum.

[19] Gingras A.C.K.S., O'Leary M.A., Sonenberg N., Hay N. (1998). 4E-BP1, a repressor of mRNA translation, is phosphorylated and inactivated by the Akt(PKB) signaling pathway. Genes Dev..

[20] Gingras AC G.S., Raught B., Polakiewicz R.D., Abraham R.T., Hoekstra M.F., Aebersold R. (1999). Regulation of 4E-BP1 phosphorylation: a novel two-step mechanism. Genes Dev..

[21] Hara K., Yonezawa K., Kozlowski M.T., Sugimoto T., Andrabi K., Weng Q.P. (1997). Regulation of eIF-4E-BP1 phosphorylation by mTOR. J. Biol. Chem..

[22] Peterson RT D.B., Hardwick J.S., Schreiber S.L. (1999). Protein phosphatase 2A interacts with the 70-kDa S6 kinase and is activated by inhibition of FKBP12–rapamycinassociated protein. Proc. Natl. Acad. Sci. U. S. A..

[23] de la Parra C., Ernlund A., Alard A., Ruggles K., Ueberheide B., Schneider R.J. (2018). A widespread alternate form of cap-dependent mRNA translation initiation. Nat. Commun..

[24] Saha B., Haizel S.A., Goss D.J. (2024). Mechanistic differences in eukaryotic initiation factor requirements for eIF4GI-driven cap-independent translation of structured mRNAs. J.Biol.Chem..

[25] Braunstein S., Karpisheva K., Pola C., Goldberg J., Hochman T., Yee H. (2007). A hypoxia-controlled cap-dependent to cap-independent translation switch in breast cancer. Mol. Cell..

[26] Meric-Bernstam F.C.H., Akcakanat A., Do K.A., Lluch A., Hennessy B.T., Hortobagyi G.N. (2012). Aberrations in translational regulation are associated with poor prognosis in hormone receptor-positive breast cancer. Breast Cancer Res..

[27] Musa J., Orth M.F., Dallmayer M., Baldauf M., Pardo C., Rotblat B. (2016). Eukaryotic initiation factor 4E-binding protein 1 (4E-BP1): a master regulator of mRNA translation involved in tumorigenesis. Oncogene.

[28] She Q.B., Halilovic E., Ye Q., Zhen W., Shirasawa S., Sasazuki T. (2010). 4E-BP1 is a key effector of the oncogenic activation of the AKT and ERK signaling pathways that integrates their function in tumors. Cancer Cell.

[29] Elena Martın M., Clara Redondo M.I.P., Isabel Álvarez M., Salinas M., Fando J.L. (2000). 4E-binding protein 1 expression is inversely correlated to the progression of gastrointestinal cancers. Inter. J. Biochem. Cell Biol..

[30] Salehi Z., Mashayekhi F. (2006). Expression of the eukaryotic translation initiation factor 4E (eIF4E) and 4E-BP1 in esophageal cancer. Clin. Biochem..

[31] Seki N., Takasu T., Sawada S., Nakata M., Nishimura R., Segawa Y. (2010). Prognostic significance of expression of eukaryotic initiation factor 4E and 4E-binding protein 1 in patients with pathological stage I invasive lung adenocarcinoma. Lung Cancer.

[32] Campbell L.J.B., Griffiths D.F., Gumbleton M. (2015). Phospho-4E-BP1 and eIF4E overexpression synergistically drives disease progression in clinically confined clear cell renal cell carcinoma. Am. J. Cancer Res..

[33] Lacerda R., Menezes J., Romao L. (2017). More than just scanning: the importance of cap-independent mRNA translation initiation for cellular stress response and cancer. Cell Mol. Life. Sci..

[34] Haizel S.A., Bhardwaj U., Gonzalez R.L., Mitra S., Goss D.J. (2020). 5'-UTR recruitment of the translation initiation factor eIF4GI or DAP5 drives cap-independent translation of a subset of human mRNAs. J. Biol. Chem..

[35] Plank T.D., Kieft J.S. (2012). The structures of nonprotein-coding RNAs that drive internal ribosome entry site function. Wiley Interdiscip. Rev. RNA.

[36] Colussi T.M., Costantino D.A., Zhu J., Donohue J.P., Korostelev A.A., Jaafar Z.A. (2015). Initiation of translation in bacteria by a structured eukaryotic IRES RNA. Nature.

[37] Pestova TV K.V., Lomakin I.B., Pilipenko E.V., Shatsky I.N., Agol V.I., Hellen C.U. (2001). Molecular mechanisms of translation initiation in eukaryotes. Proc. Natl. Acad. Sci. U. S. A..

[38] Terenin I.M., Andreev D.E., Dmitriev S.E., Shatsky I.N. (2013). A novel mechanism of eukaryotic translation initiation that is neither m^7^G-cap-, nor IRES-dependent. Nucleic Acids Res..

[39] Bailey-Serres J. (1999). Selective translation of cytoplasmic mRNAs in plants. Trends Plant Sci..

[40] Rong L., Livingstone M., Sukarieh R., Petroulakis E., Gingras A.C., Crosby K. (2008). Control of eIF4E cellular localization by eIF4E-binding proteins, 4E-BPs. RNA.

[41] Strudwick S.,B.K. (2002). The emerging roles of translation factor eIF4E in the nucleus. Differ.

[42] Blais J.D., Addison C.L., Edge R., Falls T., Zhao H., Wary K. (2006). Perk-dependent translational regulation promotes tumor cell adaptation and angiogenesis in response to hypoxic stress. Mol. Cell Biol..

[43] Mahe M., Rios-Fuller T., Katsara O., Schneider R.J. (2024). Non-canonical mRNA translation initiation in cell stress and cancer. NAR Cancer.

[44] Qin X., Jiang B., Zhang Y. (2016). 4E-BP1, a multifactor regulated multifunctional protein. Cell Cycle.

[45] Gingras A.C., Raught B., Gygi S.P., Niedzwiecka A., Miron M., Burley S.K. (2001). Hierarchical phosphorylation of the translation inhibitor 4E-BP1. Genes Dev..

[46] Shen X., Tomoo K., Uchiyama S., Kobayashi Y., Ishida T. (2001). Structural and thermodynamic behavior of eukaryotic initiation factor 4E in supramolecular formation with 4E-binding protein 1 and mRNA cap analogue, studied by spectroscopic methods. Chem. Pharma. Bull..

[47] Siddiqui N., Tempel W., Nedyalkova L., Volpon L., Wernimont A.K., Osborne M.J. (2012). Structural insights into the allosteric effects of 4E-BP1 on the eukaryotic translation initiation factor eIF4E. J. Mol. Biol..

[48] Culjkovic-Kraljacic B., Skrabanek L., Revuelta M.V., Gasiorek J., Cowling V.H., Cerchietti L. (2020). The eukaryotic translation initiation factor eIF4E elevates steady-state m(7)G capping of coding and noncoding transcripts. Proc. Natl. Acad. Sci. U. S. A..

[49] Coutinho de Oliveira L., Volpon L., Rahardjo A.K., Osborne M.J., Culjkovic-Kraljacic B., Trahan C. (2019). Structural studies of the eIF4E-VPg complex reveal a direct competition for capped RNA: implications for translation. Proc. Natl. Acad. Sci. U. S. A..

[50] Liu W., Zhao R., McFarland C., Kieft J., Niedzwiecka A., Jankowska-Anyszka M. (2009). Structural insights into parasite eIF4E binding specificity for m^7^G and m^2,2,7^G mRNA caps. J. Biol. Chem..

[51] Niedzwiecka A., Marcotrigiano J., Stepinski J., Jankowska-Anyszka M., Wyslouch-Cieszynska A., Dadlez M. (2002). Biophysical studies of eIF4E cap-binding protein: recognition of mRNA 5' cap structure and synthetic fragments of eIF4G and 4E-BP1 proteins. J. Mol. Biol..

[52] Osborne M.J., Volpon L., Kornblatt J.A., Culjkovic-Kraljacic B., Baguet A., Borden K.L. (2013). eIF4E3 acts as a tumor suppressor by utilizing an atypical mode of methyl-7-guanosine cap recognition. Proc. Natl. Acad. Sci. U. S. A..

[53] Ma X.M., Blenis J. (2009). Molecular mechanisms of mTOR-mediated translational control. Nat. Rev. Mol. Cell Biol..

[54] Shull AY N.S., Awan F.T., Liu J., Pei L., Bollag R.J., Salman H. (2015). RPPA-based protein profiling reveals eIF4G overexpression and 4E-BP1 serine 65 phosphorylation as molecular events that correspond with a pro-survival phenotype in chronic lymphocytic leukemia. Oncotarget.

[55] Andreev D.E., Dmitriev S.E., Terenin I.M., Prassolov V.S., Merrick W.C., Shatsky I.N. (2009). Differential contribution of the m^7^G-cap to the 5' end-dependent translation initiation of mammalian mRNAs. Nucleic Acids Res..

[56] Banerjee B., Goss D.J. (2014). Eukaryotic initiation factor (eIF) 4F binding to barley yellow dwarf virus (BYDV) 3'-untranslated region correlates with translation efficiency. J.Biol. Chem..

[57] Shatsky I.N., Dmitriev S.E., Terenin I.M., Andreev D.E. (2010). Cap- and IRES-independent scanning mechanism of translation initiation as an alternative to the concept of cellular IRESs. Mol. Cells..

[58] Andreev D.E., Dmitriev S.E., Zinovkin R., Terenin I.M., Shatsky I.N. (2012). The 5' untranslated region of Apaf-1 mRNA directs translation under apoptosis conditions via a 5' end-dependent scanning mechanism. FEBS Lett..

[59] Rousseau D., Gingras A.C., Pause A., Sonenberg N. (1997). The eIF4E-binding proteins 1 and 2 are negative regulators of cell growth. Oncogene.

[60] Mothe-Satney I.,Y.D., Fadden P., Haystead T.A., Lawrence J.C. (2000). Multiple mechanisms control phosphorylation of PHAS-I in five (S/T)P sites that govern translational repression. Mol. Cell. Biol..

[61] Thoreen C.C., Chantranupong L., Keys H.R., Wang T., Gray N.S., Sabatini D.M. (2012). A unifying model for mTORC1-mediated regulation of mRNA translation. Nature.

[62] Constantinou C., Elia A., Clemens M.J. (2008). Activation of p53 stimulates proteasome-dependent truncation of eIF4E-binding protein 1 (4E-BP1). Biol. Cell.

[63] Feoktistova K., Tuvshintogs E., Do A., Fraser C.S. (2013). Human eIF4E promotes mRNA restructuring by stimulating eIF4A helicase activity. Proc. Natl. Acad. Sci. U. S. A..

[64] Avanzino B.C., Fuchs G., Fraser C.S. (2017). Cellular cap-binding protein, eIF4E, promotes picornavirus genome restructuring and translation. Proc. Natl. Acad. Sci. U. S. A..

[65] Pause A B.G., Gingras A.C., Donzé O., Lin T.A., Lawrence J.C., Sonenberg N. (1994). Insulin-dependent stimulation of protein synthesis by phosphorylation of a regulator of 5'-cap function. Nature.

[66] Svitkin Y.V., Herdy B., Costa-Mattioli M., Gingras A.C., Raught B., Sonenberg N. (2005). Eukaryotic translation initiation factor 4E availability controls the switch between cap-dependent and internal ribosomal entry site-mediated translation. Mol. Cell Biol..

[67] Keiper B.D., Gan W., Rhoads R.E. (1999). Protein synthesis initiation factor 4G. Int. J. Biochem. Cell Biol..

[68] Brito Querido J., Sokabe M., Diaz-Lopez I., Gordiyenko Y., Fraser C.S., Ramakrishnan V. (2024). The structure of a human translation initiation complex reveals two independent roles for the helicase eIF4A. Nat. Struct. Mol. Biol..

[69] Oberer M., Marintchev A., Wagner G. (2005). Structural basis for the enhancement of eIF4A helicase activity by eIF4G. Genes Dev..

[70] Morino S.I.H., Svitkin Y.V., Pestova T.V., Sonenberg N. (2000). Eukaryotic translation initiation factor 4E (eIF4E) binding site and the middle one-third of eIF4GI constitute the core domain for cap-dependent translation, and the C-terminal one-third functions as a modulatory region. Mol. Cell Biol..

[71] Sokabe M., Fraser C.S. (2017). A helicase-independent activity of eIF4A in promoting mRNA recruitment to the human ribosome. Proc. Natl. Acad. Sci. U. S. A..

[72] Fletcher C.M., McGuire A.M., Gingras A.-C., Li H., Matsuo H., Sonenberg N. (1998). 4E-binding proteins inhibit the translation factor eIF4E without folded structure. Biochem.

[73] Peter D., Igreja C., Weber R., Wohlbold L., Weiler C., Ebertsch L. (2015). Molecular architecture of 4E-BP translational inhibitors bound to eIF4E. Mol. Cell..

[74] Saha B., Bhardwaj U., Goss D.J. (2023). Thermodynamically favorable interactions between eIF4E binding domain of eIF4GI with structured 5'-untranslated regions drive cap-independent translation of selected mRNAs. Biochem..

[75] Modrak-Wojcik A., Gorka M., Niedzwiecka K., Zdanowski K., Zuberek J., Niedzwiecka A. (2013). Eukaryotic translation initiation is controlled by cooperativity effects within ternary complexes of 4E-BP1, eIF4E, and the mRNA 5' cap. FEBS Lett..

[76] Pelletier J., Sonenberg N. (1988). Internal initiation of translation of eukaryotic mRNA directed by a sequence derived from poliovirus RNA. Nature.

[77] Johnson A.G., Grosely R., Petrov A.N., Puglisi J.D. (2017). Dynamics of IRES-mediated translation. Philos. Trans. R. Soc. Lond. B. Biol. Sci..

[78] Stein I.I.A., Einat P., Skaliter R., Grossman Z., Keshet E. (1998). Translation of vascular endothelial growth factor mRNA by internal ribosome entry: implications for translation under hypoxia. Mol. Cell Biol..

[79] Pestova TV S.I., Hellen C.U. (1996). Functional dissection of eukaryotic initiation factor 4F: the 4A subunit and the central domain of the 4G subunit are sufficient to mediate internal entry of 43S preinitiation complexes. Mol. Cell Biol..

[80] Jaafar Z.A., Kieft J.S. (2019). Viral RNA structure-based strategies to manipulate translation. Nat. Rev. Microbiol..

[81] Fraser C.S., Pain V.M., Morley S.J. (1999). Cellular stress in Xenopus kidney cells enhances the phosphorylation of eukaryotic translation initiation factor eIF4E and the association of eIF4F with poly(A)-binding protein. Biochem. J..

[82] Ayuso M.I., Hernandez-Jimenez M., Martin M.E., Salinas M., Alcazar A. (2010). New hierarchical phosphorylation pathway of the translational repressor eIF4E-binding protein 1 (4E-BP1) in ischemia-reperfusion stress. J. Biol. Chem..

[83] Batool A., Aashaq S., Andrabi K.I. (2019). Eukaryotic initiation factor 4E (eIF4E): a recap of the cap-binding protein. J. Cell Biochem..

[84] Beretta L.S.Y., Sonenberg N. (1996). Rapamycin stimulates viral protein synthesis and augments the shutoff of host protein synthesis upon picornavirus infection. J. Virol..

[85] Ohayon S., Yitzhaky A., Hertzberg L. (2020). Gene expression meta-analysis reveals the up-regulation of CREB1 and CREBBP in brodmann area 10 of patients with schizophrenia. Psychiatry Res..

[86] Shabani M., Sherafati Moghadam M., Moghaddami K. (2021). Effect of 8 weeks of endurance training on S6K1 and 4E-BP1 proteins content in the left ventricle of the heart of diabetic rats induced by streptozotocin and nicotinamide. J. Shahid Sadoughi Uni. Med. Sci..

[87] Badura M., Braunstein S., Zavadil J., Schneider R.J. (2012). DNA damage and eIF4G1 in breast cancer cells reprogram translation for survival and DNA repair mRNAs. Proc. Natl. Acad. Sci. U. S. A..

[88] Pilipenko E.V., Pestova T.V., Kolupaeva V.G., Khitrina E.V., Poperechnaya A.N., Agol V.I., Hellen C.U. (2000). A cell cycle-dependent protein serves as a template-specific translation initiation factor. Genes Dev..

[89] Kaminski A., Jackson R.J. (1998). The polypyrimidine tract binding protein (PTB) requirement for internal initiation of translation of cardiovirus RNAs is conditional rather than absolute. RNA.

[90] De Gregorio E.P.T., Hentze M.W. (1999). Translation driven by an eIF4G core domain in vivo. EMBO J..

[91] Clarkson B.K., Gilbert W.V., Doudna J.A. (2010). Functional overlap between eIF4G isoforms in Saccharomyces cerevisiae. PLoS One.

[92] Park E.H., Zhang F., Warringer J., Sunnerhagen P., Hinnebusch A.G. (2011). Depletion of eIF4G from yeast cells narrows the range of translational efficiencies genome-wide. BMC Genomics.

[93] De Benedetti A., Rhoads R.E. (1990). Overexpression of eukaryotic protein synthesis initiation factor 4E in HeLa cells results in aberrant growth and morphology. Proc. Natl. Acad. Sci. U. S. A..

[94] Koromilas A.E., Lazaris-Karatzas A., Sonenberg N. (1992). mRNAs containing extensive secondary structure in their 5′ non-coding region translate efficiently in cells over-expressing initiation factor eIF4E. EMBO J..

[95] Enriquez-Flores S., De la Mora-De la Mora J.I., Flores-Lopez L.A., Cabrera N., Fernandez-Lainez C., Hernandez-Alcantara G. (2022). Improved yield, stability, and cleavage reaction of a novel tobacco etch virus protease mutant. Appl. Microbiol. Biotechnol..

