# Supplementary material for: 4E-BP1 differentially regulates translation of a subset of human mRNAs
Source: J Biol Chem. 2025 Nov 26;302(1):110976. doi: 10.1016/j.jbc.2025.110976 (PMC12774752; doi:10.1016/j.jbc.2025.110976)
Supplement: Supplementary Material [file mmc1.docx]

**Supporting Information**

**4E-BP1 differentially regulates translation of a subset of human mRNAs**

Mehreen Mahbub^1,2^, Baishakhi Saha^1^ and Dixie J. Goss ^1,2,3*^

*^1^Department of Chemistry, Hunter College, City University of New York, New York, NY 10065*

*^2^Ph.D. Program in Biochemistry, The Graduate Center of the City University of New York, New York, NY,10016*

*^3^Ph.D. Program in Chemistry, The Graduate Center of the City University of New York, New York, NY 10016*

*** Corresponding author: Dixie J. Goss

Email: [dgoss@hunter.cuny.edu](mailto:dgoss@hunter.cuny.edu)

**Table S1: DNA sequences used to generate mRNA for this study**

| **5’ UTRs** | **Sense strand DNA** |
| --- | --- |
| HIF-1α | 5’-AGTGCACAGTGCTGCCTCGTCTGAGGGGACAGGAGGATCACCCTCTTCGTCGCTTCGGCCAGTG  GTCGGGCTGGGCCCTGACAAGCCACCTGAGGAGAGGCTCGGAGCCGGGCCCGGACCCCGGCGATTGCCGCCCGCTTCTCTCTAGTCTCACGAGGGGTTTCCCGCCTCGCACCCCCACCTCTGGACTTGCCTTTCCTTCTCTTCTCCGCGTGTGGAGGGAGCCAGCGCTTAGGCCGGAGCGAGCCTGGGGGCCGCCCGCCGTGAAGACATCGCGGG GACCGATTCACC-3’ |
| FGF-9 | 5’GAAACAGCAGATTACTTTTATTTATGCATTTAATGGATTGAAGAAAAGAACCTTTTTTTTTCTCTCTCTCTCTGCAACTGCAGTAAGGGAGGGGAGTTGGATATACCTCGCCTAATATCTCCTGGGTTGACACCATCATTATTGTTTATTCTTGTGCTCCAAAAGCCGAGTCCTCTG-3’ |
| p53_A_ | 5’TCTAGAGCCACCGTCCAGGGAGCAGGTAGCTGCTGGGCTCCGGGGACACTTTGCGTTCGGGCTG  GGAGCGTGCTTTCCACGACGGTGACACGCTTCCCTGGATTGGCAGCCAGACTGCCTTCCGGGTCACTGCC-3’ |
| p53_B_ | 5’-ATGGAGGAGCCGCAGTCAGATCCTAGCGTCGAGCCCCCTCTGAGTCAGGAAACATTTTCAGAC  CTATGGAAACTACTTCCTGAAAACAACGTTCTGTCCCCCTTGCCGTCCCAAGCA-3’ |
| β-actin | 5’-GGACCGCCGAGACCGCGTCCGCCCCGCGAGCACAGAGCCTCGCCTTTGCCGATCCGCCGCCCG  CCACACCCGCCGCCAGCTCAC-3’ |

**
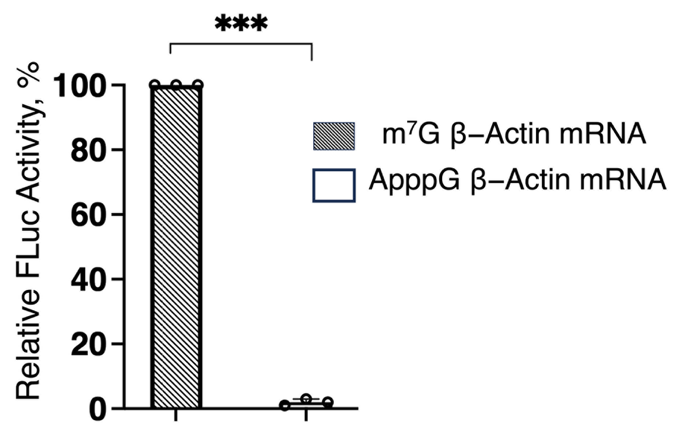
**

**Figure S1.** **4E-BP1 effects on the translation yields of m^7^G- and ApppG-capped-5’UTR-Luc mRNAs.** Comparison of translation yields of m^7^G- (-
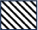
-) and ApppG-capped (-
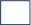
-) mRNA transcripts of β-actin.


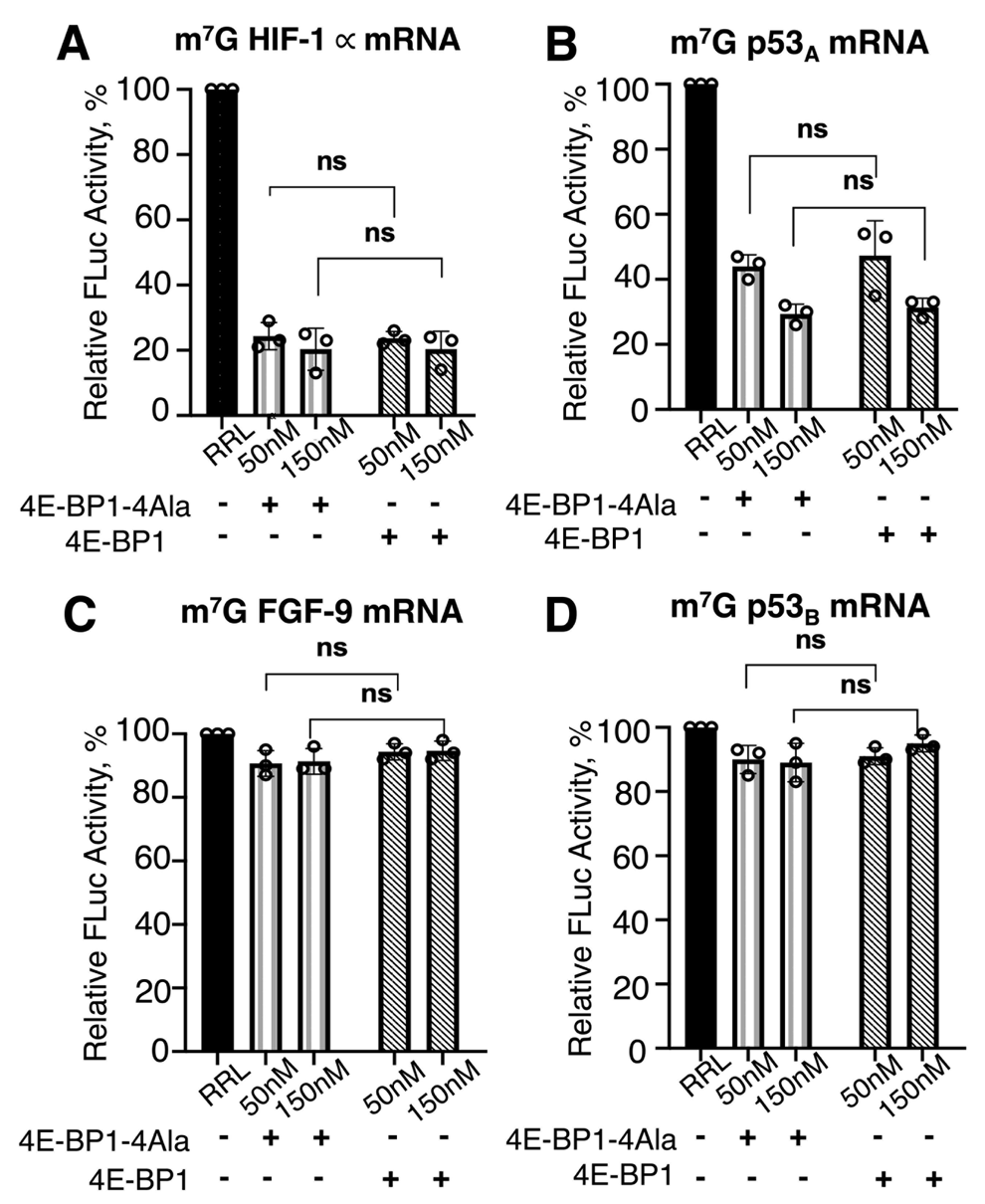


**Figure S2.** **Comparison of** **4E-BP1-4Ala versus 4E-BP1 effects on the translation yields of m^7^G-capped-5’UTR-Luc mRNAs.** Translation yields of m^7^G-capped transcripts of **(A)** HIF-1α, **(B)** p53_A_, **(C)** FGF-9, and **(D)** p53_B_-UTR-Luc mRNAs following treatment of the RRL with increasing concentrations of either the non-phosphorylatable 4E-BP1-4Ala mutant or wild-type 4E-BP1. **Vertically striped bar** (-
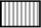
-), represents translation in the presence of 4E-BP1-4Ala; diagonally **striped bar** (-
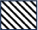
-), represents translation in the presence of 4E-BP1. Bar heights and error bars correspond to the average and standard deviations, respectively, of three independent luciferase activity measurements with control RRL (without 4E-BP1) set at 100%. Data were analyzed by two-tailed unpaired Student’s t-test, where p represents the probability that differences occurred by chance: n.s, p =0.12; *, p <0.033; **, p =0.002; ***, p < 0.001.

**
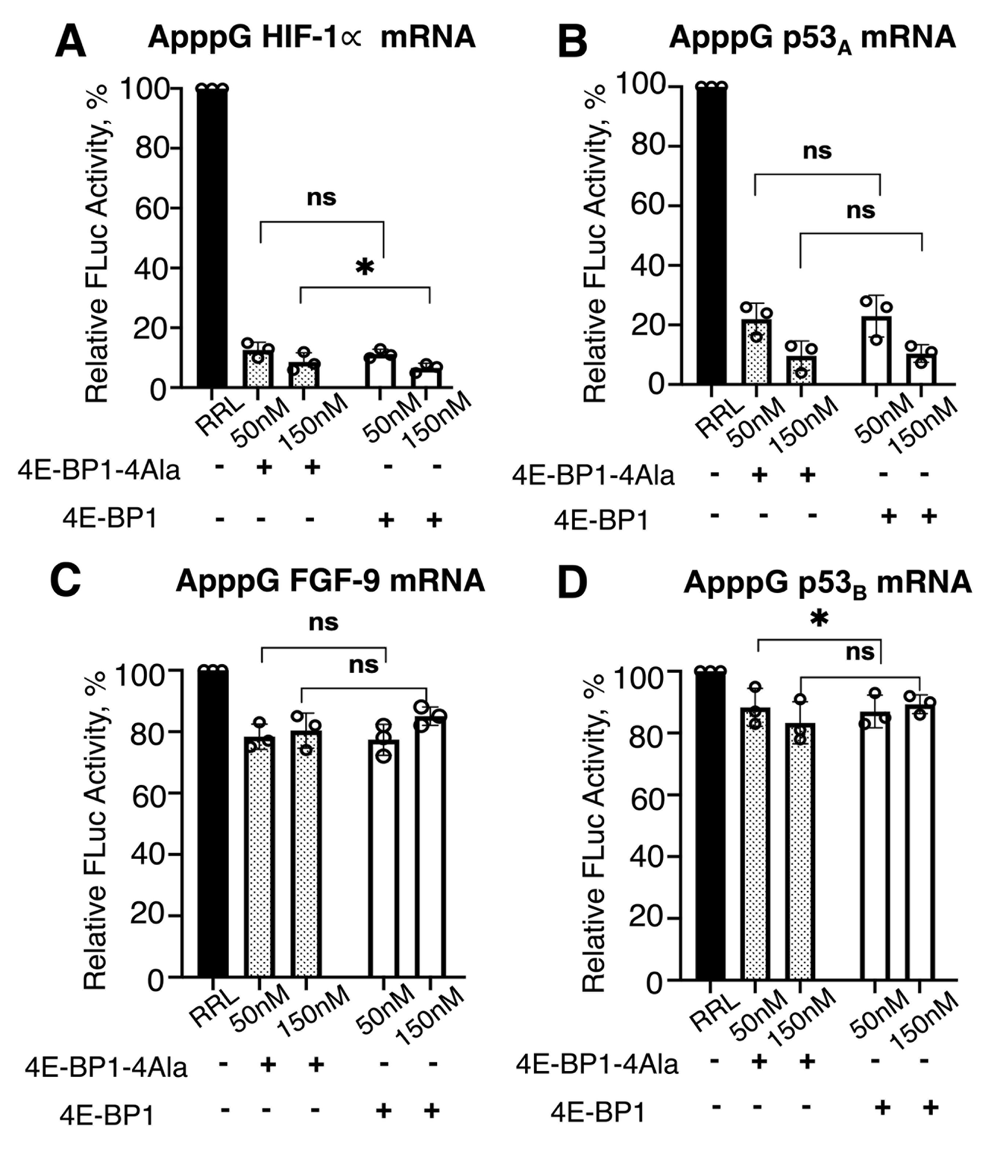
**

**Figure S3.** **Comparison of** **4E-BP1-4Ala versus 4E-BP1 effects on the translation yields of ApppG-capped-5’UTR-Luc mRNAs.** Translation yields of ApppG-capped transcripts of **(A)** HIF-1α, **(B)** p53_A_, **(C)** FGF-9, and **(D)** p53_B_-UTR-Luc mRNAs following treatment of the RRL with increasing concentrations of either the non-phosphorylatable 4E-BP1-4Ala mutant or wild-type 4E-BP1. **Dotted bar** (-
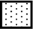
-), represents translation in the presence of 4E-BP1-4Ala; white **bar** (-
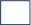
-), represents translation in the presence of 4E-BP1. Bar heights and error bars correspond to the average and standard deviations, respectively, of three independent luciferase activity measurements with control RRL (without 4E-BP1) set at 100%. Data were analyzed by two-tailed unpaired Student’s t-test, where p represents the probability that differences occurred by chance: n.s, p =0.12; *, p < 0.033; **, p =0.002; ***, p < 0.001.

**
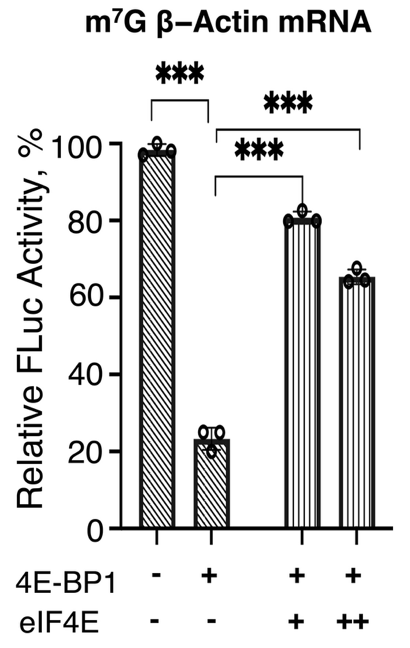
**

**Figure S4:** **Effect of eIF4E on the translation yields of m^7^G-capped β-actin-5’UTR-Luc mRNA transcripts.** Translation yields of control m^7^G-capped β-actin-UTR-Luc mRNA, following treatment of the RRL with 50 nM of 4E-BP1 and increasing concentrations of eIF4E. Diagonally **striped bar** (-
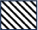
-) represents translation in the presence of 4E-BP1 alone; **vertically striped bar** (-
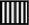
-) represents increasing concentrations of eIF4E in the presence of 4E-BP1 (**+**, 50 nM; **++**, 150 nM eIF4E). 4E-BP1 was held constant at 50 nM in all conditions. Relative luciferase activity was normalized to the respective controls (m^7^G-capped-UTR-Luc mRNA) for each reporter constructs with no 4E-BP1 added to RRL. Bar heights and error bars correspond to the average and standard deviations, respectively, of three independent luciferase activity measurements. Data were analyzed by two-tailed unpaired Student’s t-test, where p represents the probability that differences occurred by chance: n.s, p =0.12; *, p < 0.033; **, p =0.002; ***, p < 0.001.

**
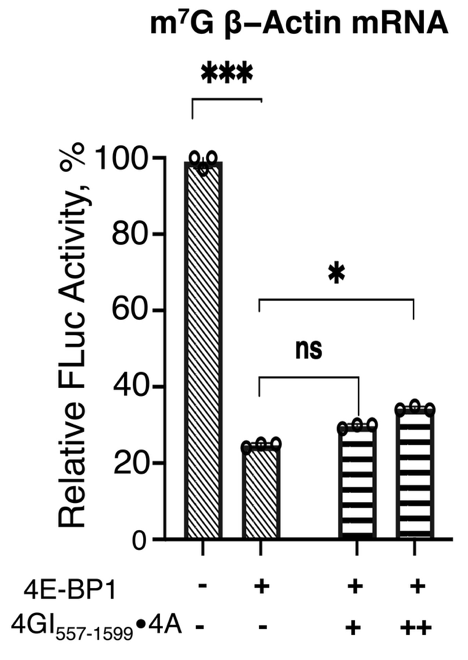
**

**Figure S5:** **Effect of eIF4GI_557-1599_**•**eIF4A on the translation yields of m^7^G-capped β-actin-5’UTR-Luc mRNA transcripts.** Translation yields of control m^7^G-capped β-actin-UTR-Luc mRNA, following treatment of the RRL with 50 nM of 4E-BP1 and increasing concentrations of eIF4GI_557-1599_•eIF4A. Diagonally **striped bar** (-
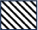
-) represents translation in the presence of 4E-BP1 alone; **horizontally striped bar** (-
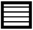
-) represents increasing concentrations of eIF4GI_557-1599_•eIF4A in the presence of 4E-BP1 (**+**, 50 nM; **++**, 150 nM eIF4GI_557-1599_•eIF4A). 4E-BP1 was held constant at 50 nM in all conditions. Relative luciferase activity was normalized to the respective controls (m^7^G-capped-UTR-Luc mRNA) for each reporter constructs with no 4E-BP1 added to RRL non-depleted of eIF4GI_557-1599_ or eIF4A. Bar heights and error bars correspond to the average and standard deviations, respectively, of three independent luciferase activity measurements. Data were analyzed by two-tailed unpaired Student’s t-test, where p represents the probability that differences occurred by chance: n.s, p =0.12; *, p < 0.033; **, p =0.002; ***, p < 0.001.

**
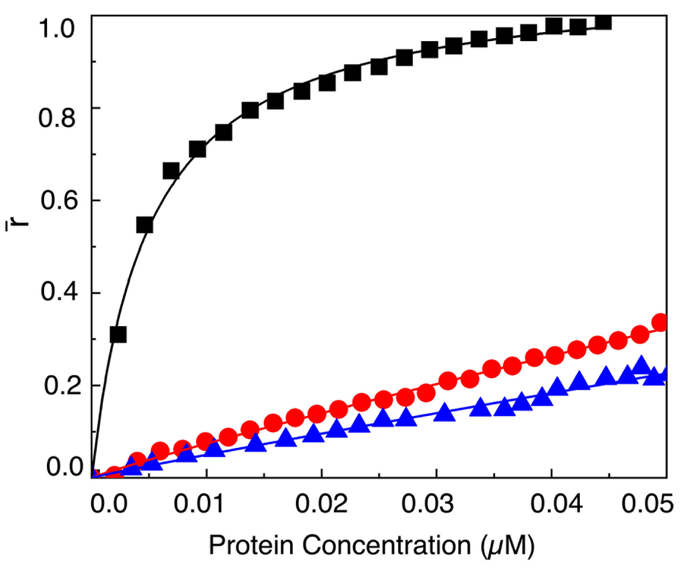
**

**Figure S6: Fluorescence anisotropy analysis of eIF4GI_557-1599_ binding to eIF4E•4E-BP1-FITC-Peptide complex.** Normalized anisotropy changes (denoted as r̄) for the interaction of eIF4E binding to 4E-BP1-FITC-Peptide (-■-), eIF4GI_557-1599_ binding to 4E-BP1-FITC-Peptide (-●-), and eIF4GI_557-1599_ binding to eIF4E**•**4E-BP1-FITC-Peptide complex (-▲-) **using 20 nM fluorescein-labeled 4E-BP1 peptide**. Data points corresponding to the average from three independent anisotropy measurements were normalized and plotted against protein concentration ($\mu$M). Curves represent Hill-equation fits, as deciphered in materials and methods, used to calculate the corresponding K_D_ values.


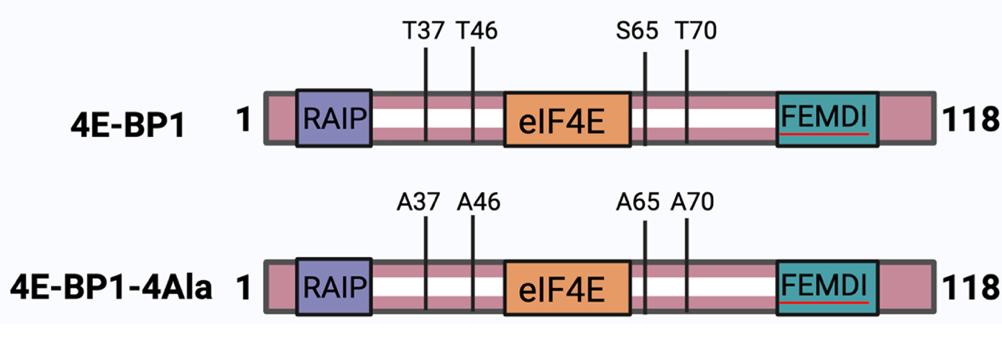


**Figure S7:** Domain organization of the 4E-BP1 and **4E-BP1-4Ala.**


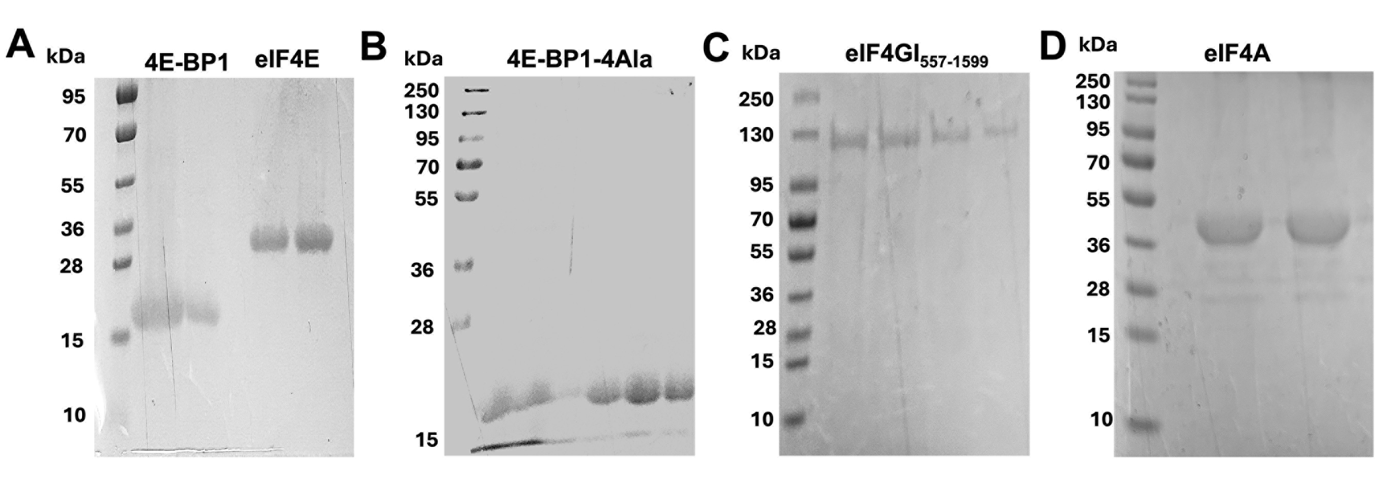


**Fig. S8:** Images of 10% SDS-PAGE gels with elution containing (**A**) 4E-BP1, and eIF4E, (**B**) 4E-BP1-4Ala, (**C**) eIF4GI_557-1599_ and (**D**) eIF4A respectively from using Ni-NTA column, Heparin columns and Ni-NTA spin column respectively.
